# Supplementary material for: PIKfyve inhibition induces LRRK2 activation through phosphatidylinositol 3-phosphate and CASM
Source: Life Sci Alliance. 2026 Nov 6;10(1):e202603939. doi: 10.26508/lsa.202603939 (PMC13743929; doi:10.26508/lsa.202603939)

Figure 1C

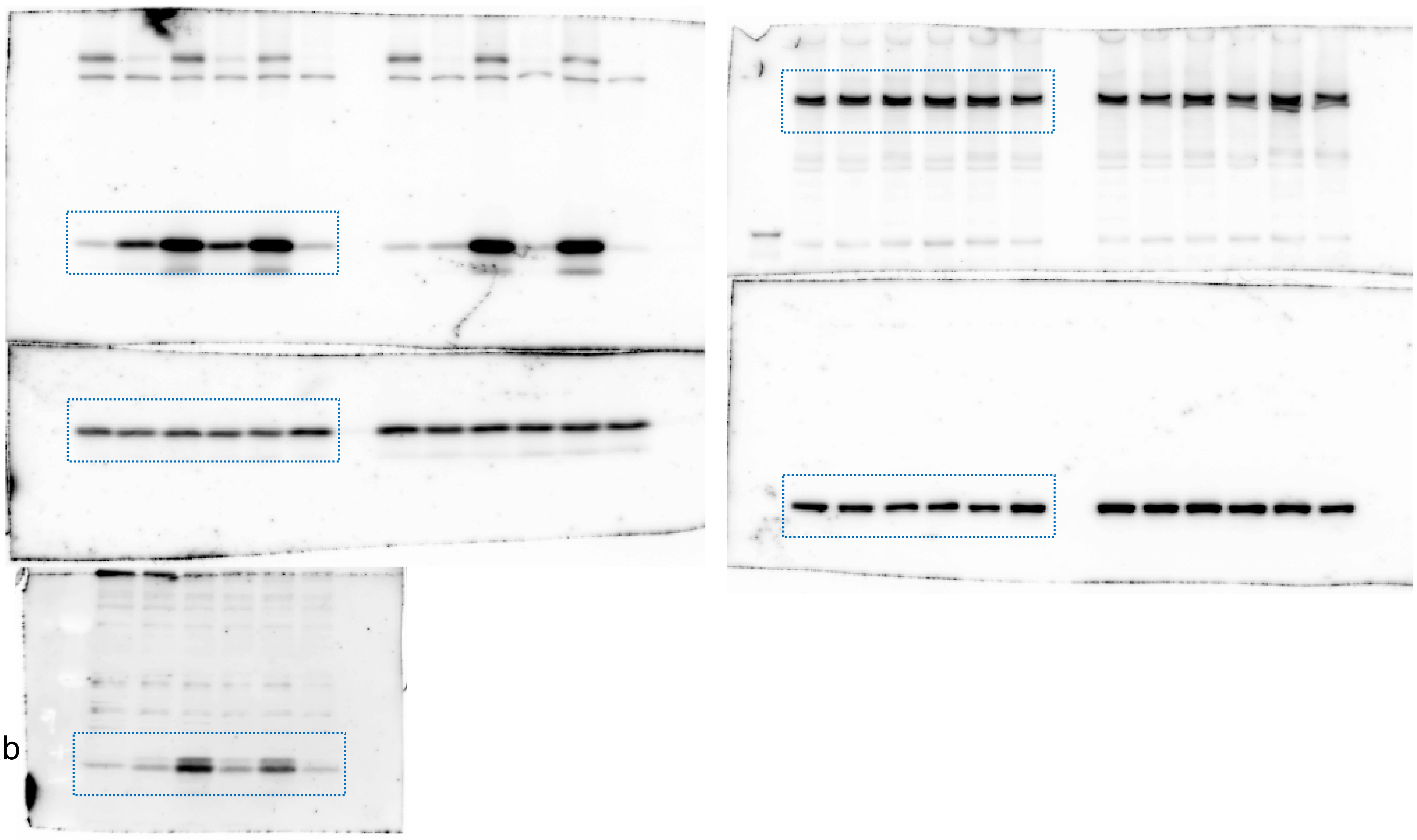

Figure 1E

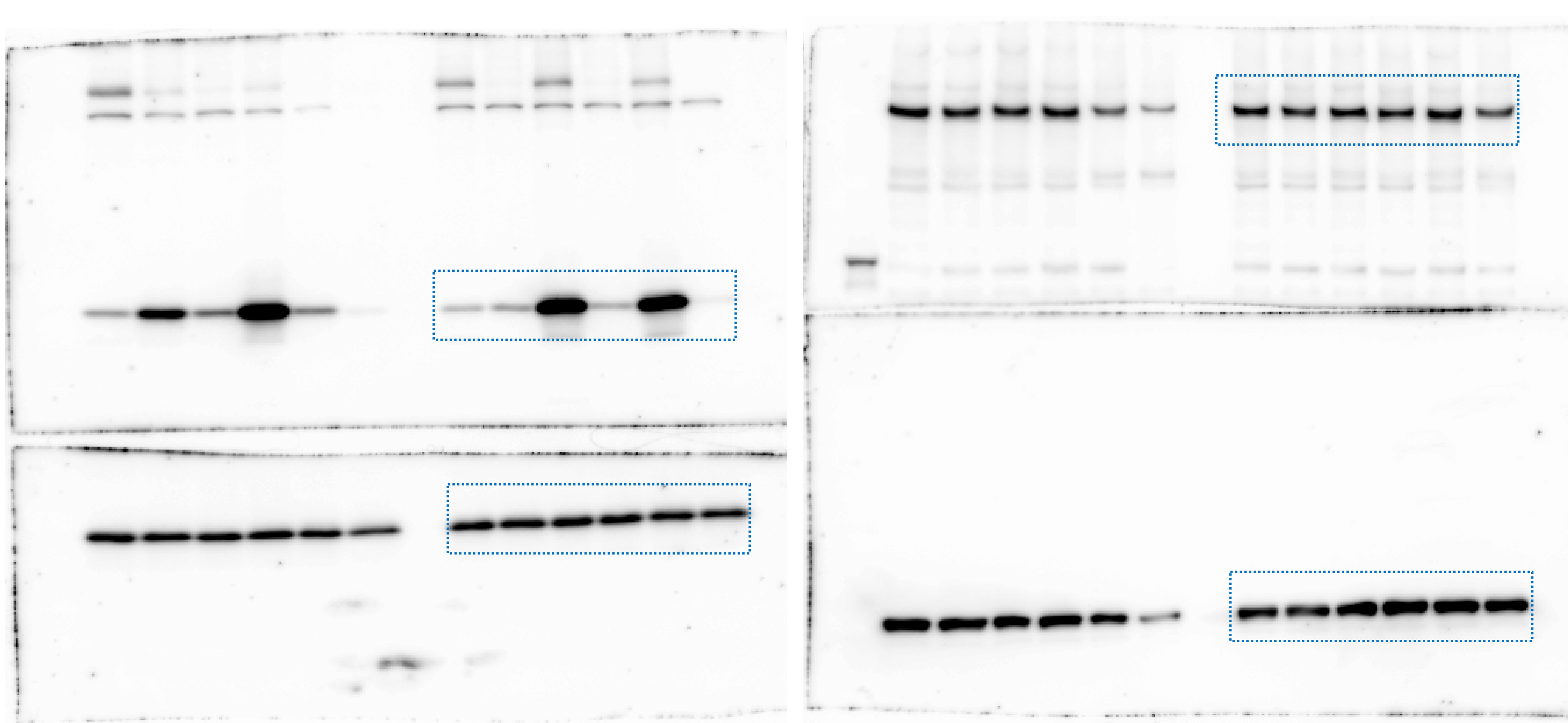

Figure 1G

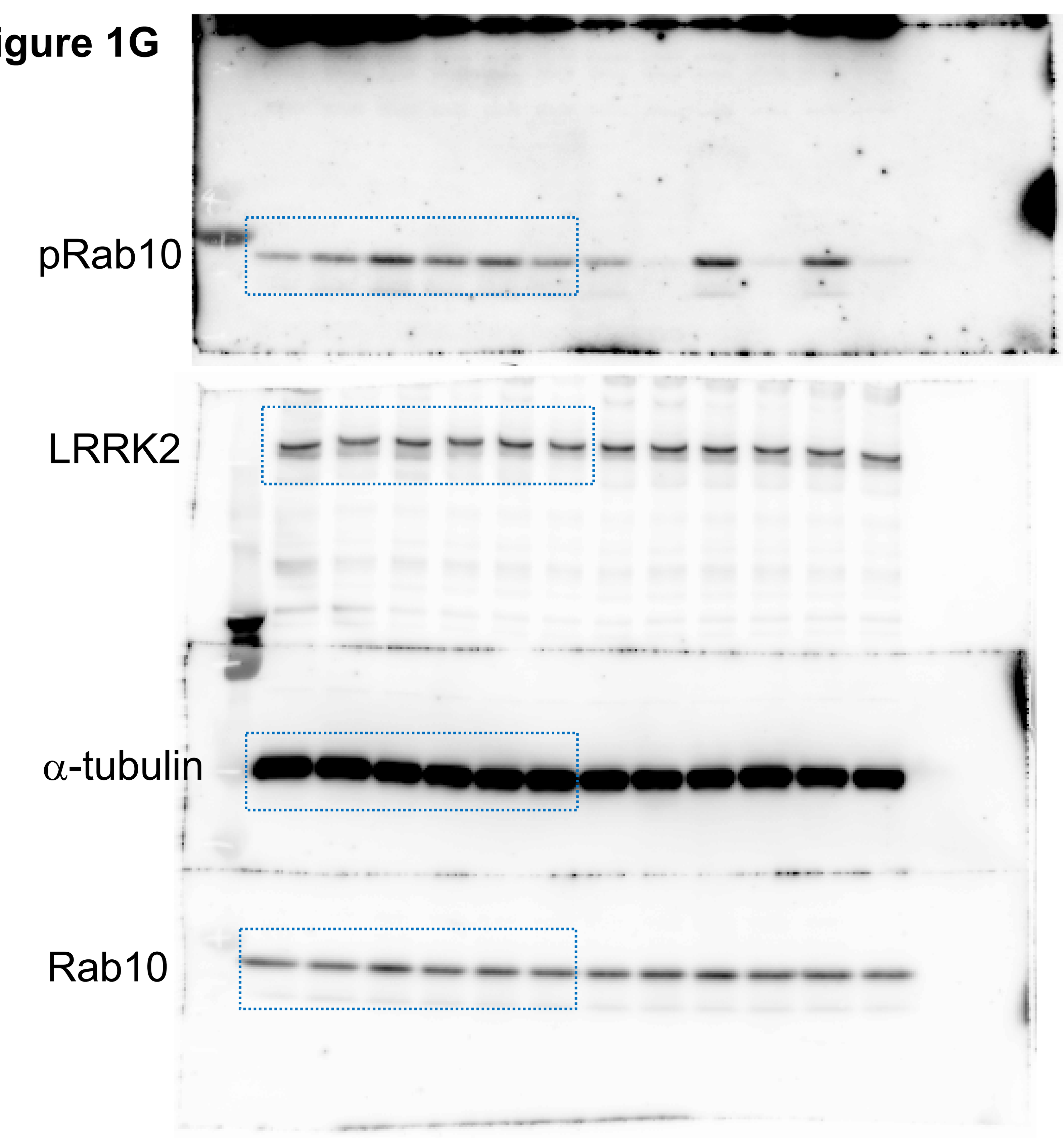

Figure 1I

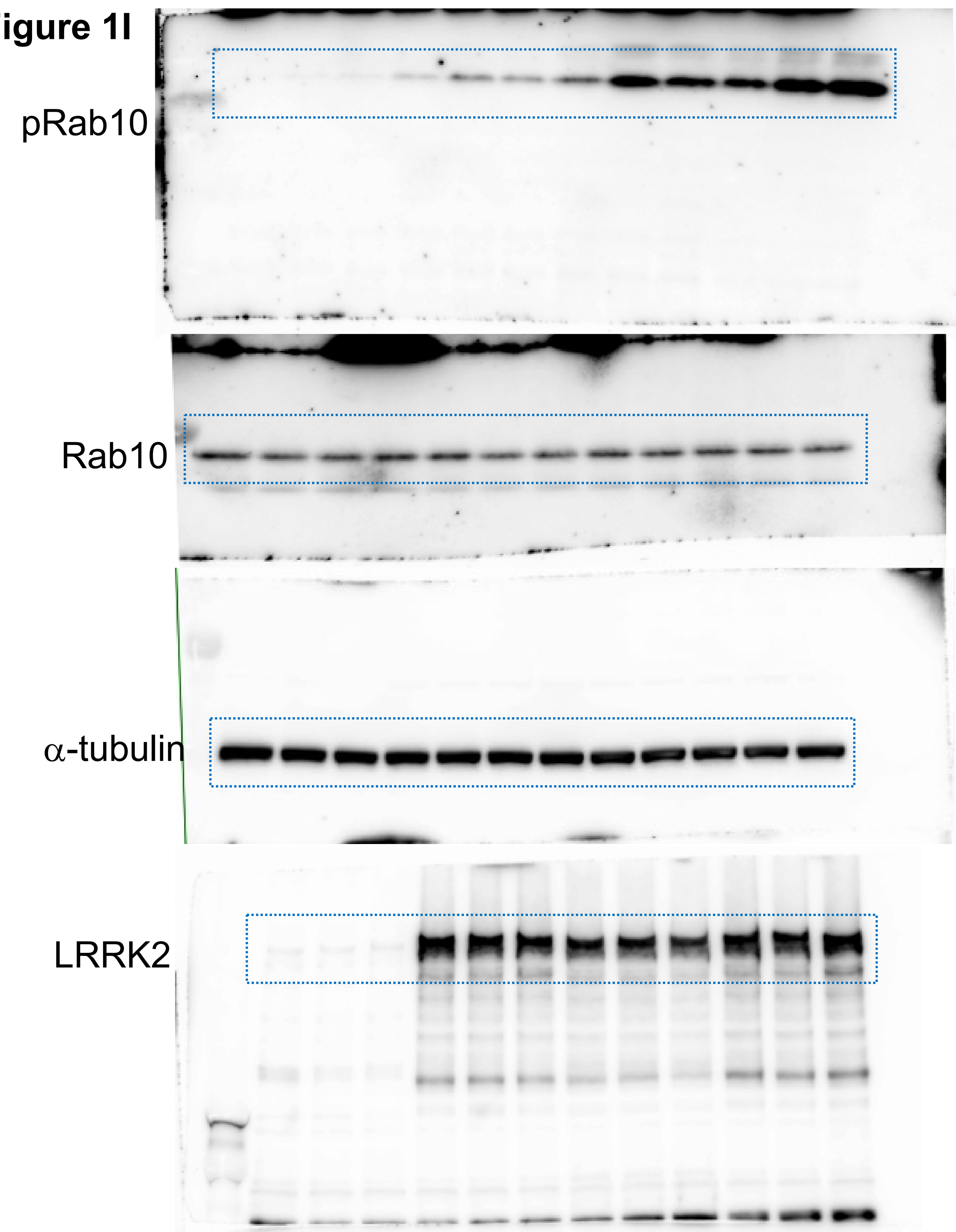

Figure 1L

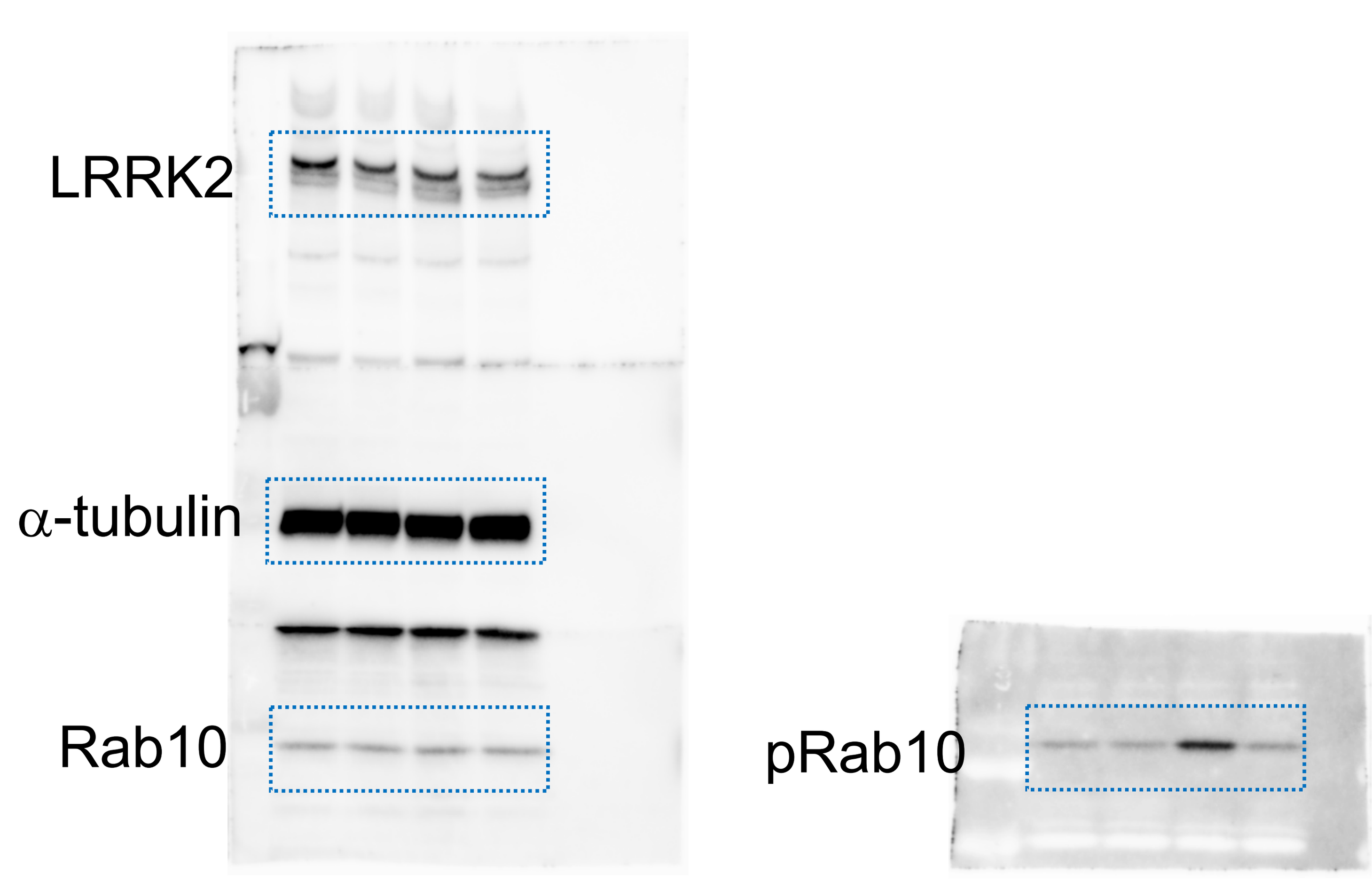

Supplement: Supplementary file 1 [file LSA-2026-03939_SdataF1.pdf]
