# Supplementary material for: PIKfyve inhibition induces LRRK2 activation through phosphatidylinositol 3-phosphate and CASM
Source: Life Sci Alliance. 2026 Nov 6;10(1):e202603939. doi: 10.26508/lsa.202603939 (PMC13743929; doi:10.26508/lsa.202603939)

Figure 2A, F, G

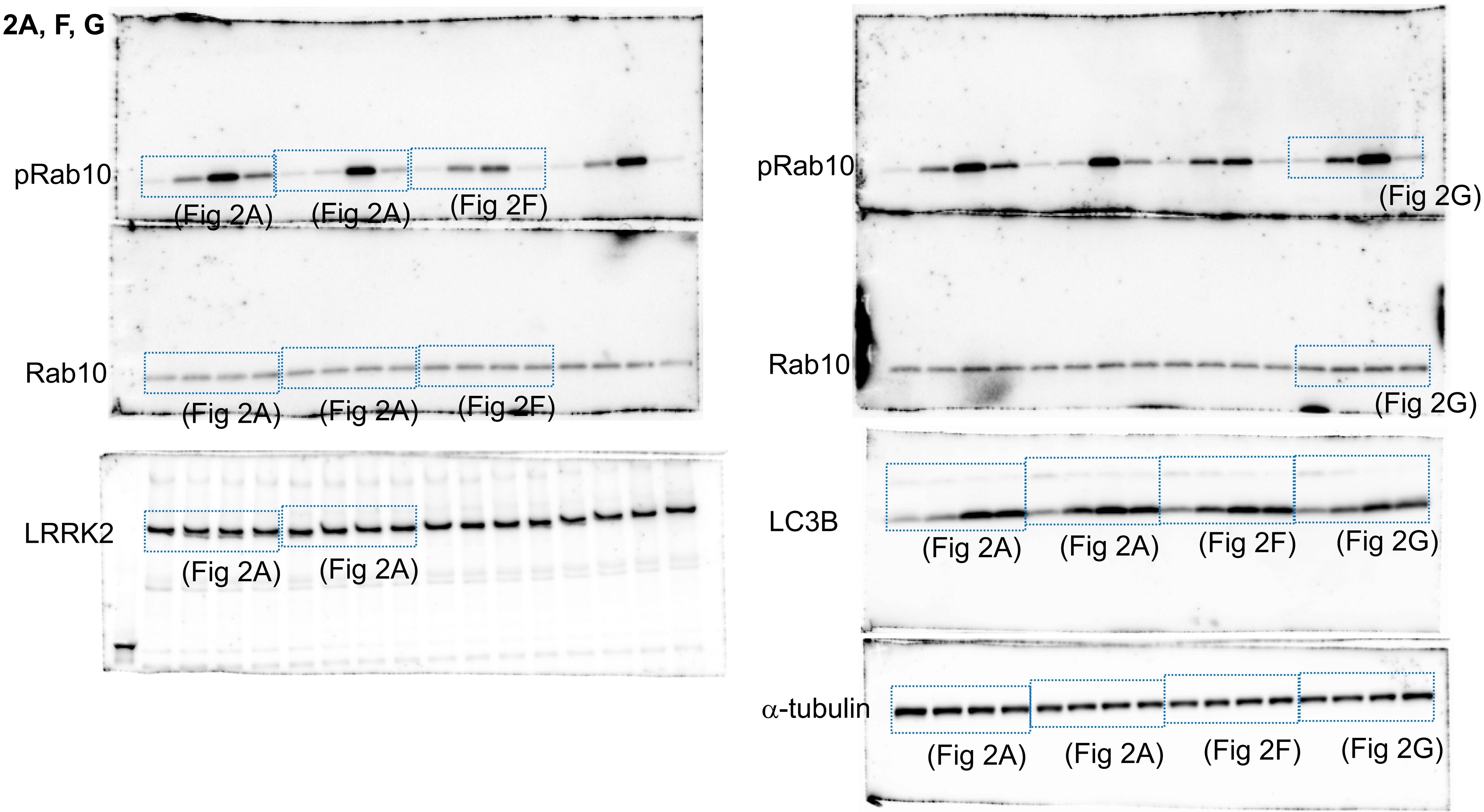

Figure 2F

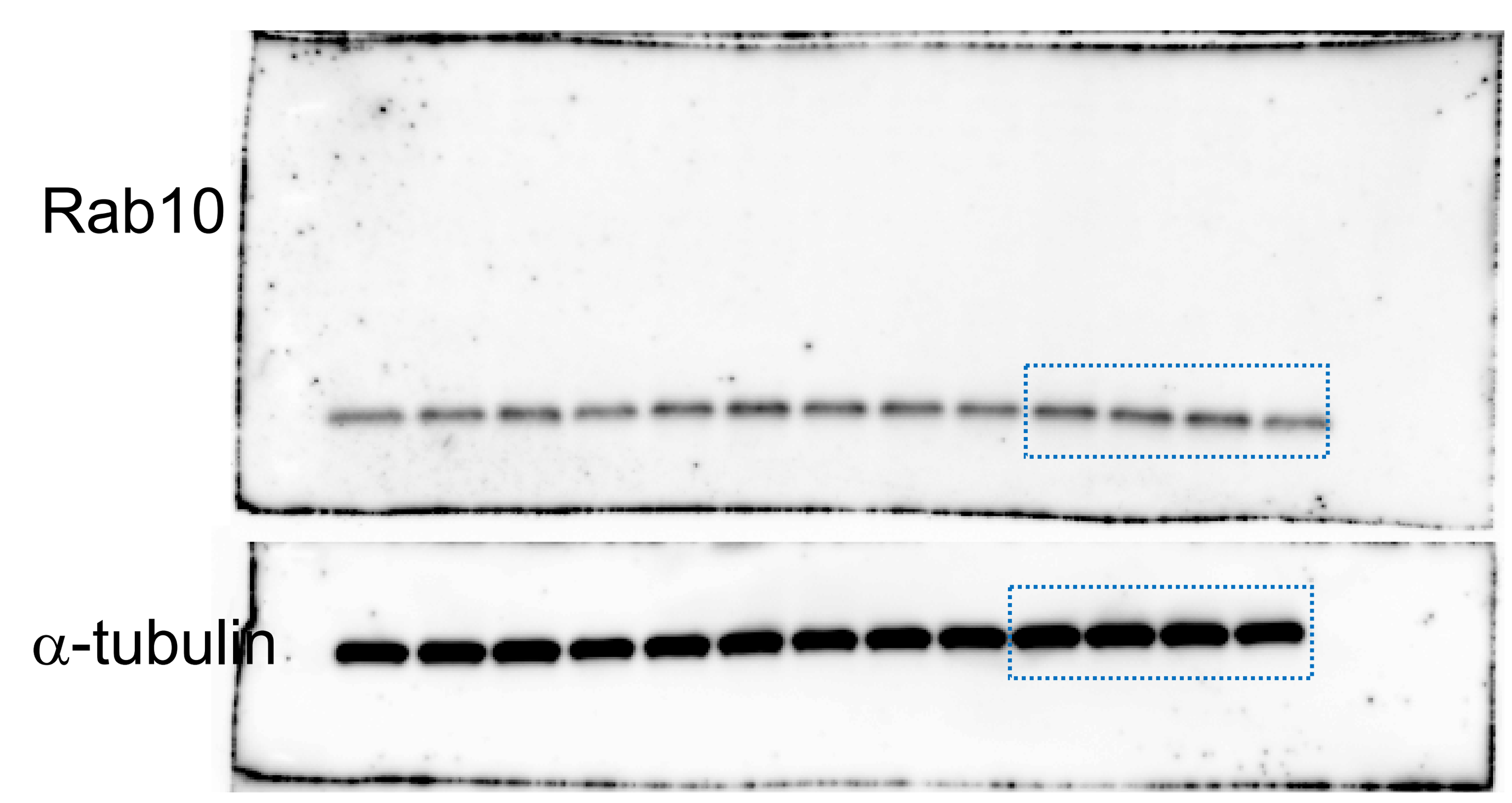

Figure 2G

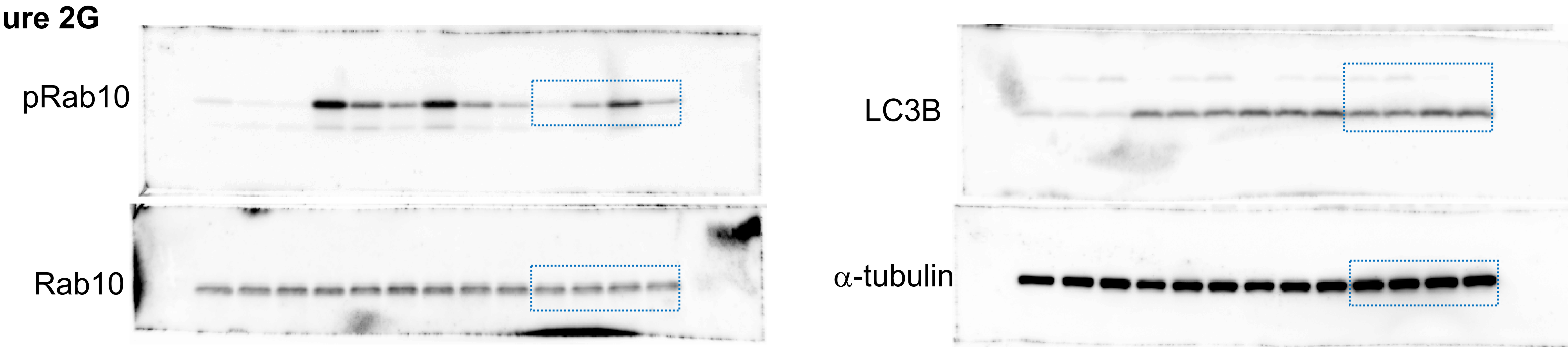

Supplement: Supplementary file 2 [file LSA-2026-03939_SdataF2.pdf]
