# Supplementary material for: PIKfyve inhibition induces LRRK2 activation through phosphatidylinositol 3-phosphate and CASM
Source: Life Sci Alliance. 2026 Nov 6;10(1):e202603939. doi: 10.26508/lsa.202603939 (PMC13743929; doi:10.26508/lsa.202603939)
Supplement: Supplementary file 3 [file LSA-2026-03939_SdataF3.pdf]

Figure 3B

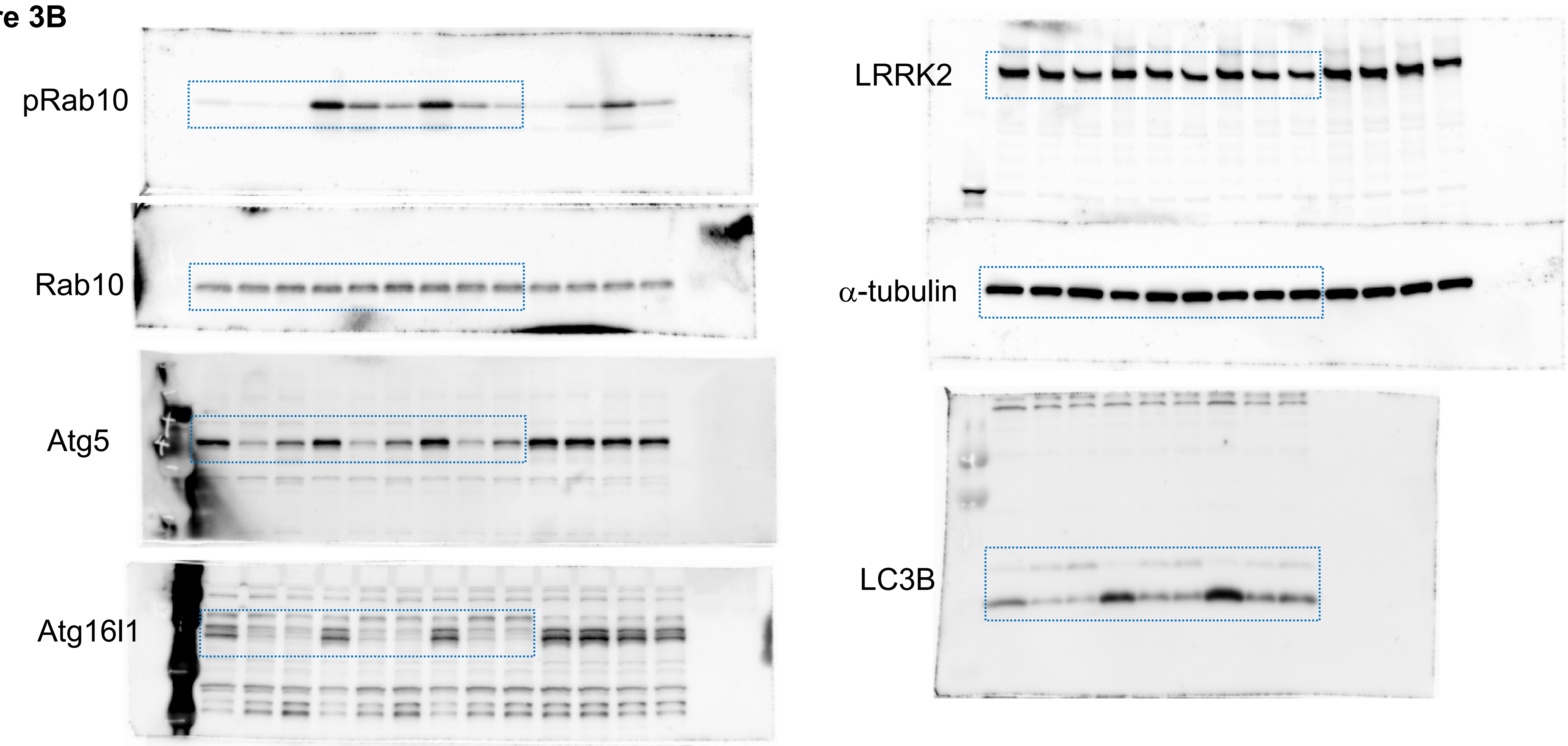

Figure 3D

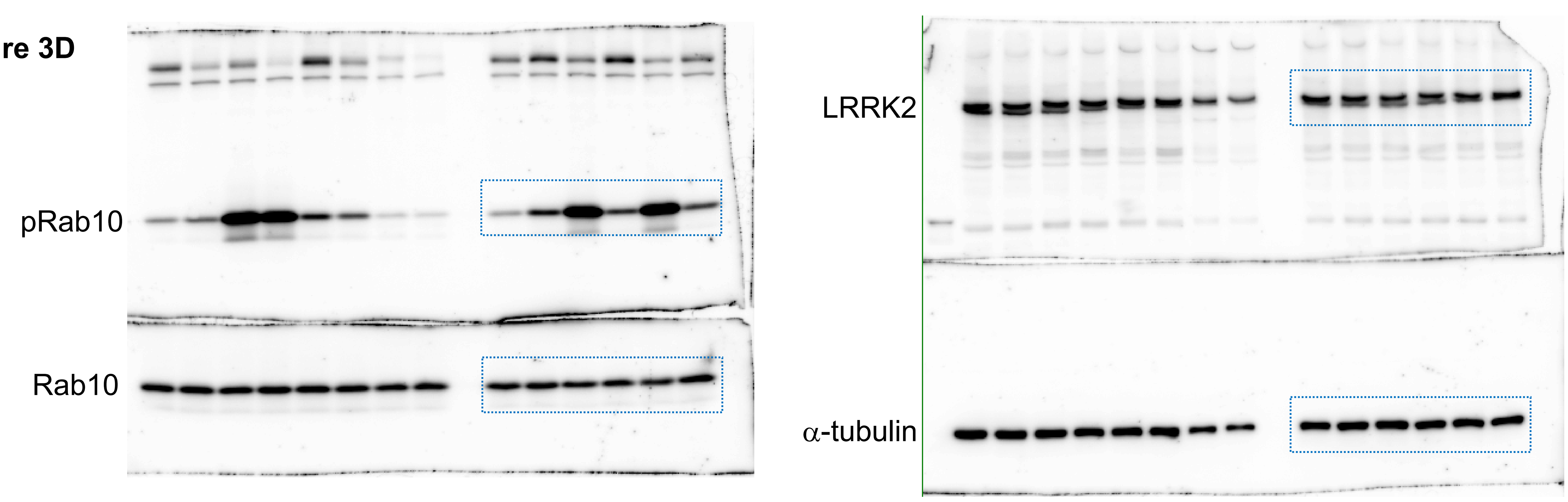

Figure 3F

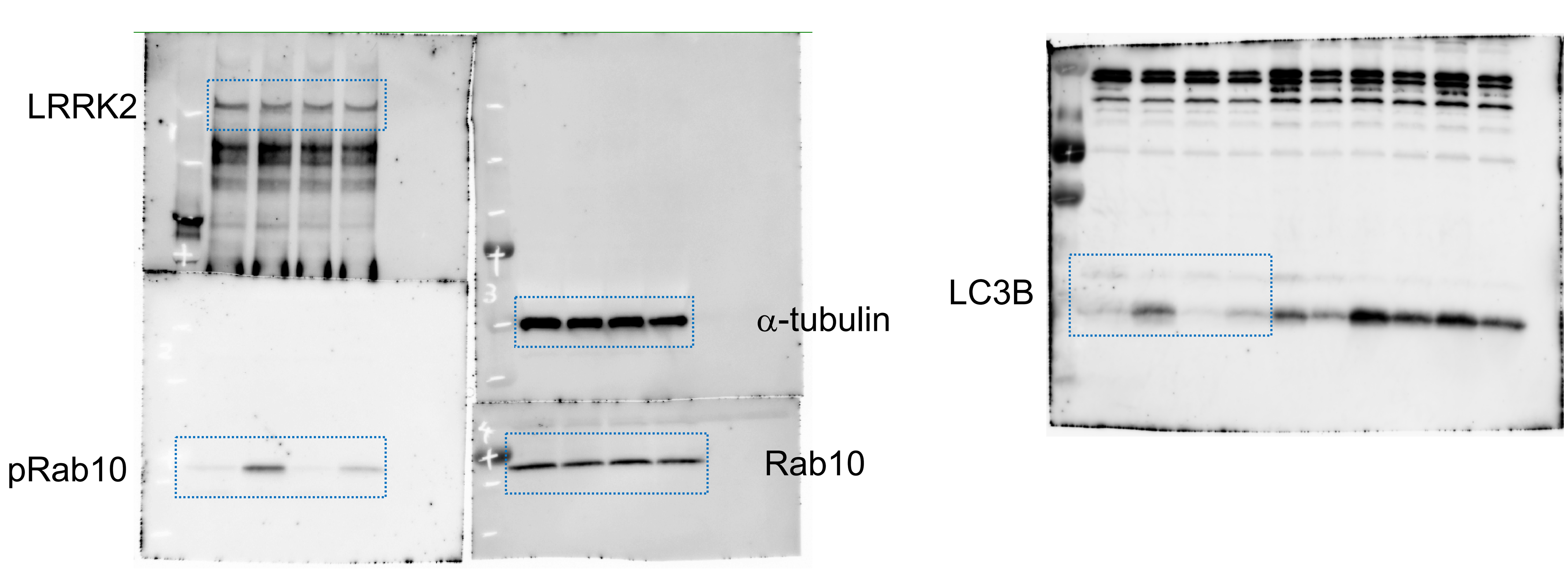

Figure 3H

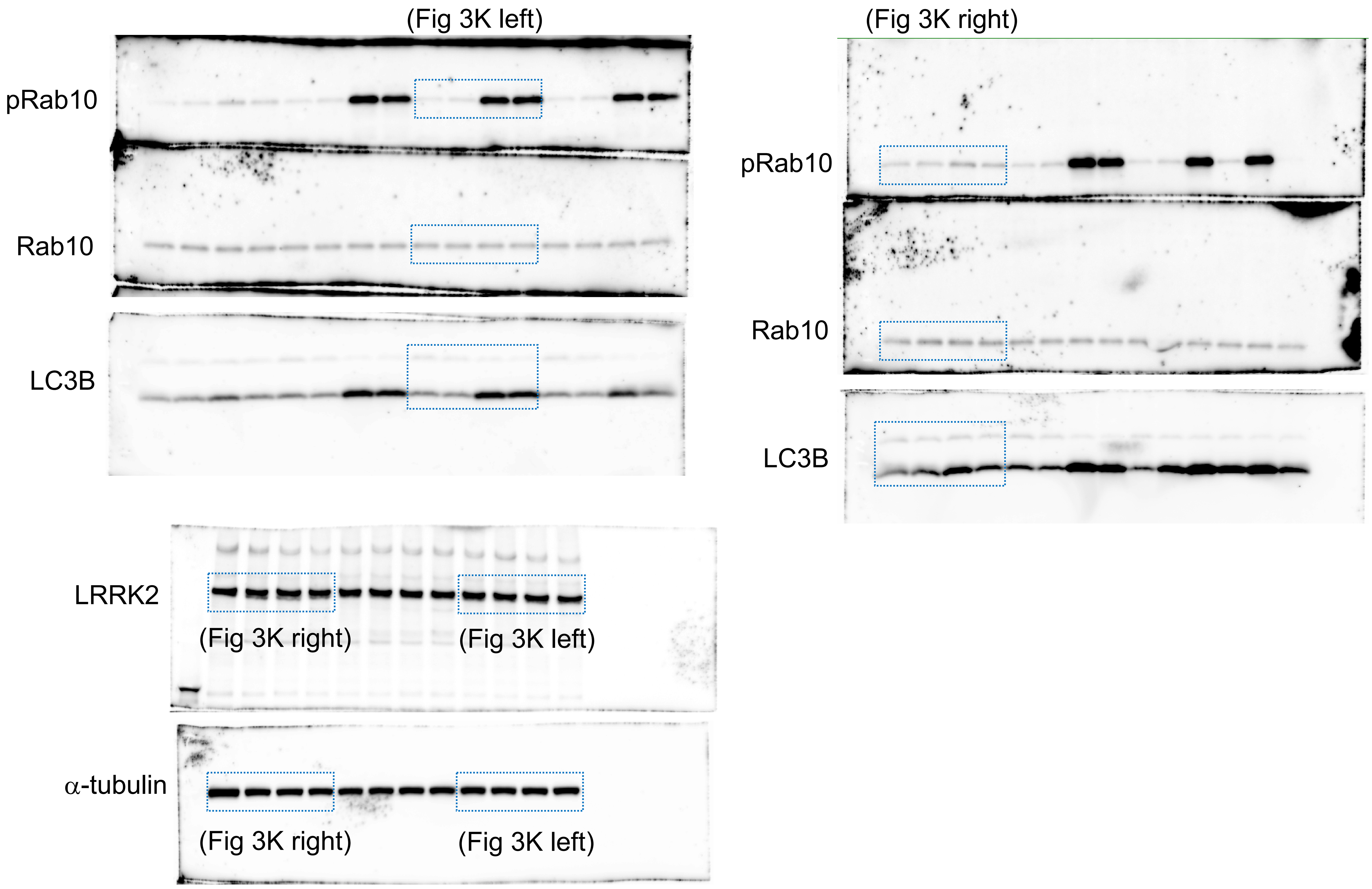
(Fig 3K left)
(Fig 3K right)
pRab10
Rab10
LC3B
pRab10
Rab10
LC3B
LRRK2
(Fig 3K right)
(Fig 3K left)
α-tubulin
(Fig 3K right)
(Fig 3K left)
