# Supplementary material for: PIKfyve inhibition induces LRRK2 activation through phosphatidylinositol 3-phosphate and CASM
Source: Life Sci Alliance. 2026 Nov 6;10(1):e202603939. doi: 10.26508/lsa.202603939 (PMC13743929; doi:10.26508/lsa.202603939)

Figure S1A

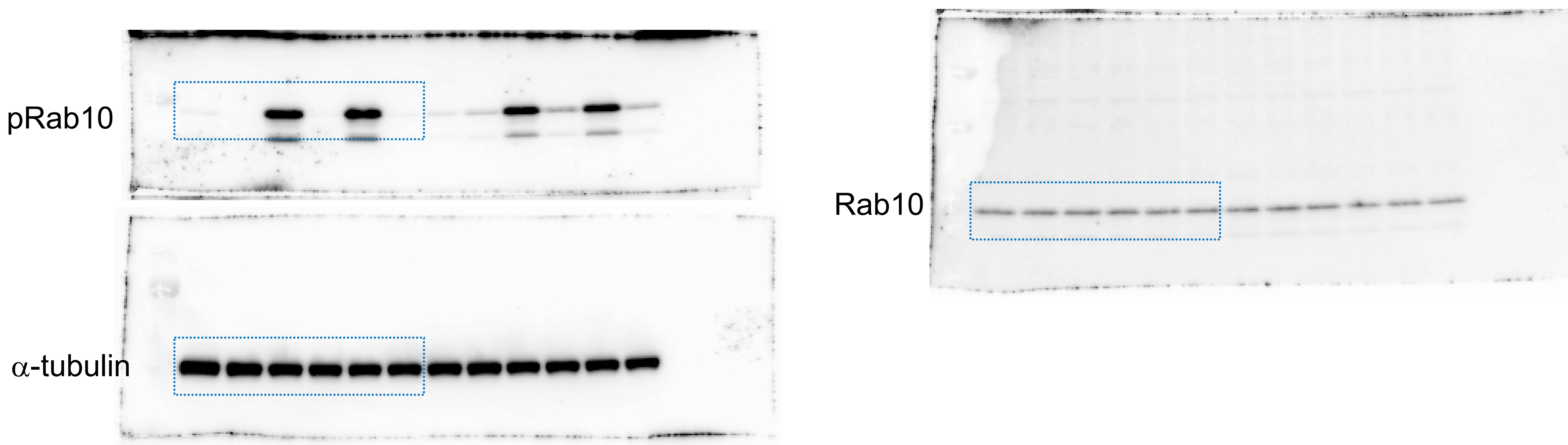

Figure S1B

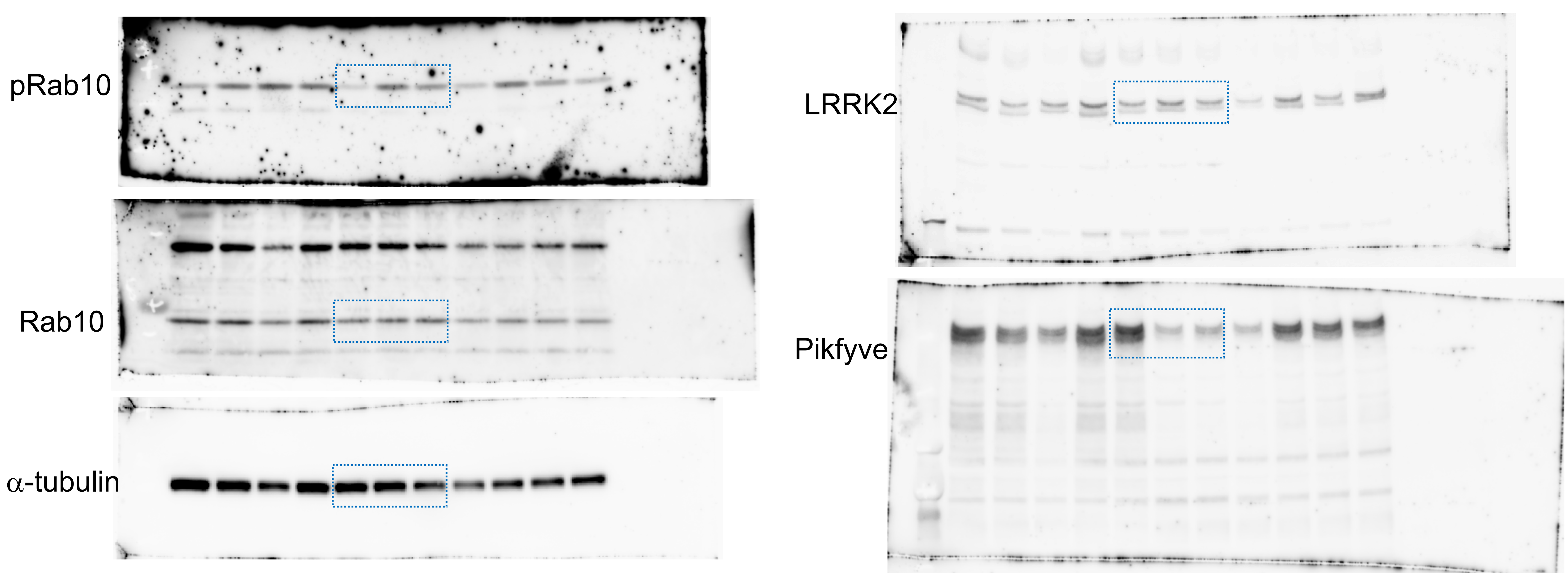

Figure S1D

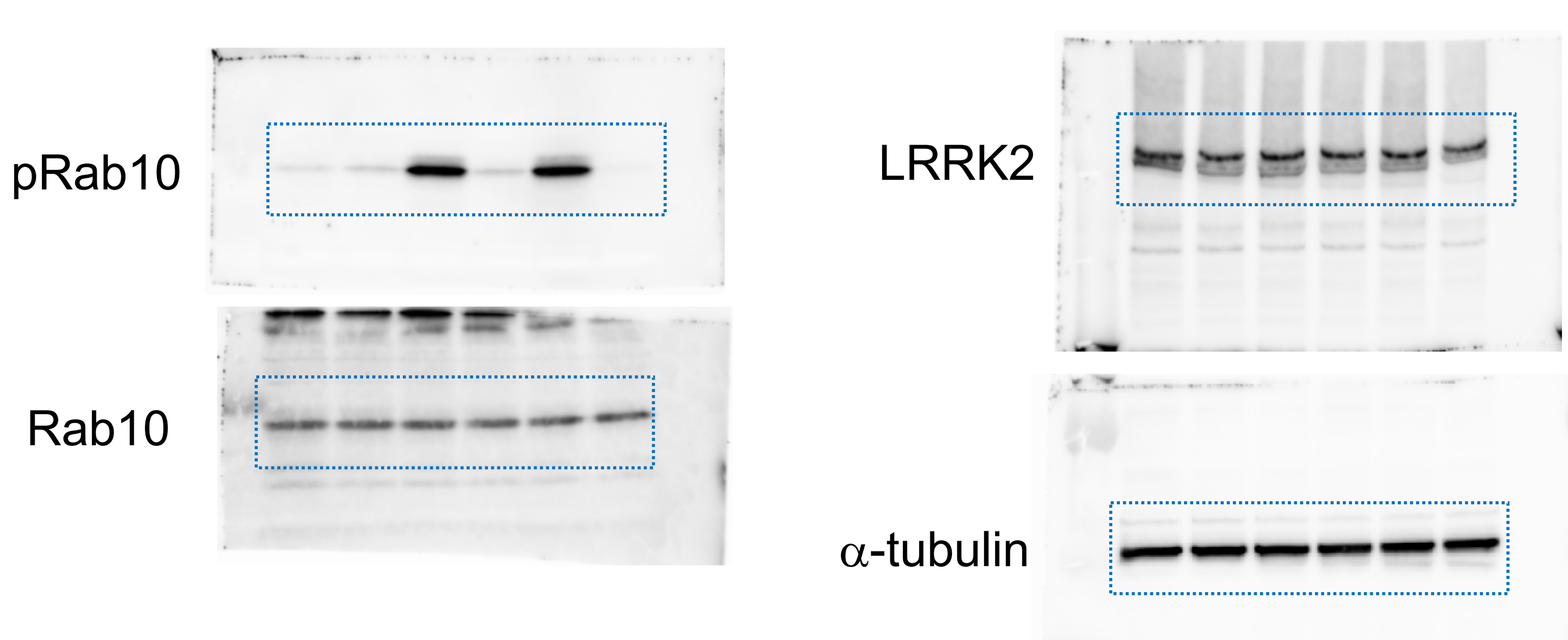

Figure S1F

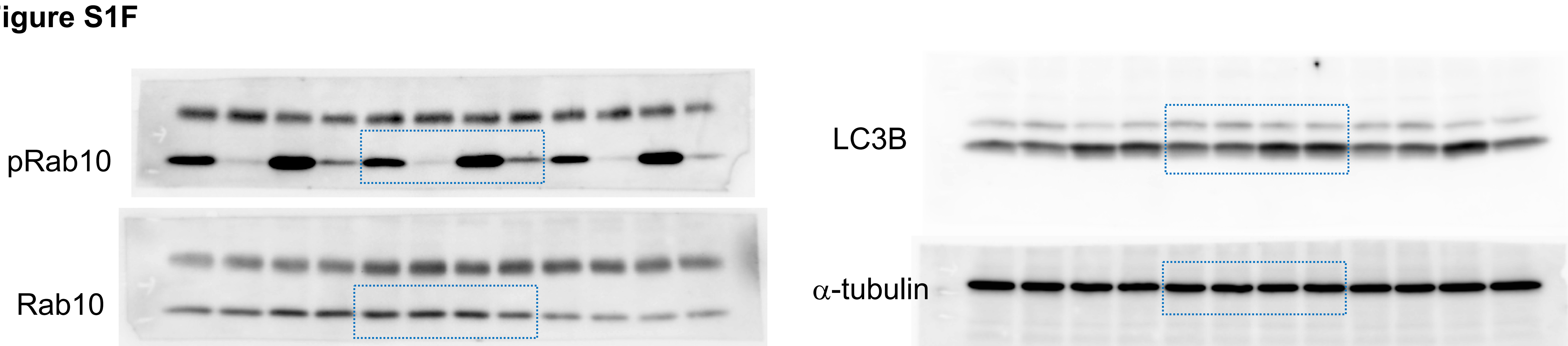

Supplement: Supplementary file 6 [file LSA-2026-03939_SdataFS1.pdf]
