# Supplementary material for: PIKfyve inhibition induces LRRK2 activation through phosphatidylinositol 3-phosphate and CASM
Source: Life Sci Alliance. 2026 Nov 6;10(1):e202603939. doi: 10.26508/lsa.202603939 (PMC13743929; doi:10.26508/lsa.202603939)
Supplement: Supplementary file 9 [file LSA-2026-03939_review_history.pdf]

PIKfyve inhibition induces LRRK2 activation through phosphatidylinositol 3-phosphate and CASM

Tomoki Kuwahara, Gen Yoshii, Maria Sakurai, Shoichi Suenaga, Hiroki Nakanishi, Matthew Jefferson, Thomas Wileman, Kentaro Tomii, and Takeshi Iwatsubo
DOI: https://doi.org/10.26508/lsa.202603939

Corresponding author(s): Tomoki Kuwahara, The University of Tokyo

Review Timeline:

Submission Date:	2026-10-17
Editorial Decision:	2026-10-21
Revision Received:	2026-10-27
Accepted:	2026-10-28

Scientific Editor: Tim Fessenden

Transaction Report:

(Note: With the exception of the correction of typographical or spelling errors that could be a source of ambiguity, letters and reports are not edited. The original formatting of letters and referee reports may not be reflected in this compilation.)

Please note that the manuscript was previously reviewed at another journal and the reports were taken into account in the decision-making process at *Life Science Alliance*.

Reviews

Review #1

Comments to the Authors (Required):
The authors have tried to respond to the reviewer comments but several issues have not yet been resolved.

1. My main concern relates to the data that leads the authors to conclude that LRRK2 binds PI3P. Dot blots are notorious for providing spurious results. The authors needed to mutate 5 residues to see a decrease in pRab10 (6F) that they attribute to loss of PI3P binding. But the data have not been corrected for the lower amounts of LRRK2 in the cells expressing those proteins, and that mutant seems lower even with no PI3P increase in the controls.
They show a binding assay with no inputs or means to interpret efficiency or use of the penta A mutant or a specific positive control (Fig. 5B), with no significant difference between PI3P and PI3,5P2. They show a "PIPosome kinase assay" in Fig. S5 where there is no phosphoinositide selectivity in regard to stimulating LRRK2 autophosphorylation (A) or pRab10 increase with increasing PI3P. Thus the title does not seem to be supported by the data shown thus far.
2. Other issues:
A. As I stated originally, inhibiting PI3P with VPS34-IN1 should decrease LRRK2 activity if PI3P is a LRRK2 activator. In panel 1C, there is a huge increase in pRab10 with this inhibitor in the absence of Api; two other inhibitors, less so, but lack of influence on LRRK2 activity supports my concern. The ability of Api to activate LRRK2 seems to require the presence of PI3P so is decreased when both agents are present. This needs to be stated explicitly, rather than swept under the rug.
B. Page 8. The authors use a method for PI3,5P2 measurement but refer to a reference that does not compare apilomod to control. As an example, Shimanaka et al. (2022) https://doi.org/10.1038/s42004-022-00676-6 showed that Apilomod lowers PI3,5P2 and increases PI3P, and HPLC methods using 3H-inositol labeled cells has shown the same thing in many other studies (Weisman, Emr, etc). HPLC methods have no problem distinguishing PI3,5P2 from PI4,5P2. Thus it remains unclear why addition of a PIKfyve inhibitor would not change PI3,5P2 levels, putting the presented data in question. Reviewer #3 noted that this lipid is very rare and thus perhaps there is some issue of recovery? The authors need to explain this issue better.
C. The authors are hand waving in their rebuttal about whether CASM and apilomod are in the same pathway. If so, the abstract needs to be revised accordingly.

Review #2

Comments to the Authors (Required):
The authors have significantly improved their manuscript, addressing my major concerns around confirmation that Rab10 phosphorylation is LRRK2-dependent, inclusion of orthogonal validation of the PIKfyve inhibitor results by knockdown, validation of the endogenous LRRK2 antibody, quantification of the colocalization imaging data, and clarification of the partial CASM dependence and the novelty of the work relative to recent preprints. Two of my requests, a genetic approach to deplete VPS34 and imaging of PentaA LRRK2 localization, were not feasible for technical reasons, but the authors explain these limitations and have tempered their conclusions accordingly. I have no additional comments and feel this is appropriate for publication in this journal.

Review #1

Comments to the Authors (Required):
In this paper, Kuwahara et al. explore the role of phosphoinositides in LRRK2 kinase activation. There are currently 3 preprints out (including this submission, one other in review at this journal), showing that PI3,5 "PIKfyve" kinase inhibition causes lysosome swelling and LRRK2 activation. This team was the first to show previously that ionophores such as chloroquine and the peptide LLOME activate LRRK2. This reviewer very much appreciates the high quality of the data

herein-the gels are very beautiful and carefully generated. There are also interesting results presented but the conclusions are not yet supported by the data and some of the findings are perplexing and don't quite make sense at this stage.

1. The authors would like to conclude the PI3P activates LRRK2. Yet in figure 1C and 2B-E, in the control reactions, PI3 kinase inhibitors (depleting PI3P) activate LRRK2. So the authors cannot state that PI3P activates LRRK2 under control conditions because they show it does not. Apilomod activates LRRK2 and causes lysosome swelling but less so in the joint presence of a PI3 kinase inhibitor. In LAP type CASM, a UVRAG-Rubicon-VPS34 complex generates PI3P on phagosomes to support LC3 lipidation, so VPS34 is functionally important. In other CASM contexts, ATG16L1 is recruited via V ATPase and membrane stress, allowing CASM to proceed largely independently of VPS34. Perhaps STING activation goes through the latter route while swollen lysosomes use the former? Specific requirements for components of the various pathways should be tested for their possible roles.
2. In figure 2H, the authors measure lipid levels with various inhibitors. Apilimod blocks PI3,5P generation but no change is seen in this lipid in panel H? Why? This figure is comparing apilimod with chloroquine, monensin and nigericin which likely have very different mechanisms of LRRK2 activation. What about in a Clc7 KO cell line? [The authors should mention at least one preprint that has been out there on the same topic.]
3. Page 7. The authors suggest that STRESS-induced LRRK2 activation requires PI3P but the purpose of this lipid is not yet clear. Line 189: LRRK2 activity is positively correlated with PI3P-NOT SO in control reactions. Line 215: CASM is required for activation of LRRK2 induced by increased PI3P (not shown-perhaps: "that occurs coincident with increased PI3P").
4. Line 265. Not clear if LAMP staining surrounds the structures as the cells are full of lysosomes
5. The authors would like to test if LRRK2 binds PI3P. Dot blots are not reliable or sufficient to support this conclusion-this would require flotation of LRRK2 (and mutants) with liposomes of specific phosphoinositide composition. Line 312: not yet demonstrated.

In summary, this manuscript presents a great deal of work and carefully executed pharmacological experiments. For the story to be suitable for further consideration at this journal, the authors would need to teach us what PI3P is actually doing (or bring us closer to a pathway) to give apparent LRRK2 activation upon lysosome swelling. To this reader, it is not yet clear.

Review #2

Comments to the Authors (Required):
Summary:
Kuwahara and colleagues add new insights around the mechanisms by which lysosomal membrane damage activate LRRK2 by demonstrating that PI3P is a key requirement for LRRK2 activation following endolysosomal membrane permeabilization. Using a combination of pharmacological manipulations, they show that compounds that lead to increased PI3P levels (e.g. PIKfyve inhibition) lead to increased LRRK2 activity and suggest that this occurs in a CASM and Rab dependent manner. Overall, this work builds on previous work to increase our understanding of the signals that lead to LRRK2 recruitment to damaged endolysosomes.

While this work provides additional mechanistic clarity as to how LRRK2 activity is regulated in response to lysosomal stress, additional work is needed to further strengthen and clarify the authors' claims to warrant publication in this journal.

Main points of the manuscript and associated concerns:

1) LRRK2 activity is increased upon pharmacological manipulations that lead to increased PI3P levels:
a. The authors employ multiple pharmacological conditions (PIKfyve inhibition, inhibition of PI3K with multiple compounds) and multiple cell types (RAW macrophages, A549 cells, and HEK293 cells overexpression LRRK2) to demonstrate that conditions that increase PI3P levels lead to increased phosphorylation of Rab10. This data is very compelling.

b. However, additional work is needed to confirm that the increase in Rab10 phosphorylation is LRRK2 kinase dependent. Specifically, additional studies should be done demonstrating that LRRK2 kinase inhibition (e.g. with MLi-2) reduced Rab10 phosphorylation in response to modulating PI3P levels. They include this control in their studies using the TAT-D11 peptide but it is not included in any of their pharmacological studies in Figures 1C-J, and this data

is required to be confident that this truly leads to an increase in LRRK2 kinase activity.

c. All of the studies in Figure 1 rely on pharmacological methods for increased PI3P levels. The use of orthogonal genetic approaches to modulate PI3P levels (e.g. PIKfyve KD/KO) would also be helpful supportive evidence of their conclusions.

d. Additionally, the authors suggest that the effects reported by many in the field using compounds that damage lysosomes or perturb lysosomal homeostasis more generally lead to LRRK2 activation in a manner dependent on PI3P. These conclusions would also be further strengthened if shown using an orthogonal genetic approach in addition to treatment with VPS34-IN1 or Cmpd19 to reduced PI3P levels.

2) PI3P-mediated activation of LRRK2 requires CASM and specific Rab 12 and 29:

a. The authors show that Rab10 phosphorylation induced by PIKfyve inhibition requires key factors involved in CASM using KD of ATG16L1 and ATG5 and BMDMs from mice that express mutant Atg16l1 that lacks the WD40 domain due to a stop codon inserted after E230. This suggests that PI3P-dependent regulation of LRRK2 activity still requires the CASM machinery. They also rule out that the PI3P-Nox2 pathway upstream of CASM does not mediate LRRK2-dependent activation via PIKfyve inhibition.

b. These data overall strongly support their conclusions that PI3P-mediated activation of LRRK2 depends on CASM. In Figure 3, the genetic manipulations to inhibit CASM lead to reduced but not absent increases in pRab10 levels (the Atg5 and ATG16l1 and E230 conditions reduced pRab10 levels following apilimod treatment but do not bring it back to baseline levels). Do the authors think that additional non-CASM dependent mechanisms for inducing LRRK2 activation following apilimod treatment are occurring? Or is this due to residual CASM activity (although harder to justify in the BMDM model)? It would be helpful to add additional text or data to explain this discrepancy.

c. For their experiments assessing Rab12 and 29 knockdown (Figure 4), there is no baseline pRab10 levels in control RAW macrophage cells in the blots shown. Is this due to an exposure issue or due to no detection in this experiment? This has impacts on the quantification in terms of fold change shown in C and D and confidence in effect sizes observed.

3) LRRK2 and pRab10 levels colocalize with PI3P on lysosomes:

a. The authors perform microscopy-based studies to establish that PI3P (visualized using a genetically-encoded probe) and LRRK2 or pRab10 colocalize after treatment with apilimod, YM201535 or CQ. They show that there is increased colocalization with the PI3P-detecting probe and LRRK2/pRab10 following treatment with agents that lead to increased PI3P levels.

b. They visualize endogenous LRRK2 using an antibody; however, imaging-based assessment of endogenous LRRK2 is notoriously difficult given the available antibodies. To add confidence to this data, they should confirm that this antibody is LRRK2 selective through comparison to signal seen in LRRK2 KO or LRRK2 siRNA-treated RAW264.7 cells.

c. Additionally, they show images of colocalization between LAMP1, LRRK2, and their PI3P probe but do not provide any quantification as to whether this is increased following apilimod or CQ treatment. No untreated images are shown in panel 4E, which also makes it difficult to understand whether this is an increase observed following treatments that elevate PI3P levels. Additional controls should be included to show the staining pattern of these under baseline conditions and to quantify the colocalization following treatment.

4) PI3P regulates LRRK2 activation through direct binding via N-terminal positively charged amino acids in LRRK2:

a. The authors perform elegant studies assessing whether PI3P leads to increased LRRK2 activation by directly binding to LRRK2 and identify a cluster of positively-charged residues on the N-terminus of LRRK2 that contribute to this.

b. Their data shows that the PentaA construct in which five amino acids in this region of LRRK2 are substituted to alanine leads to reduced binding of PI3P. This proposed biochemical mechanism would be further strengthened with

additional data assessing the localization of the PentaA LRRK2 variant in cells. Based on their model, the prediction would be that LRRK2 localization to lysosomes following PIKfyve inhibition of the Penta LRRK2 variant would be reduced, and imaging-based experiments to directly assess this would add confidence that this interaction plays a role in this process through its effects on membrane recruitment of LRRK2.

5) Novelty of observations and context within existing literature:

a. The authors' data provides compelling support that PI3P is another key piece of the puzzle around how LRRK2 is activated in response to endolysosomal stress. The novelty of the work should be further articulated in the discussion, as treatment with apilimod has been previously shown to lead to LRRK2 activation. This work does significantly extent the previously published findings, but additional text referencing previous findings and articulating the novel insight from this work would be helpful in the Discussion section.

Review #3

Comments to the Authors (Required):
Summary

Kuwahara et al., set out to address the gap in knowledge regarding the role of lipids in LRRK2 kinase activity. The authors focus on phosphoinositides, finding that pharmacological inhibition of PIKfyve, the major kinase converting PI3P to PI35P2, induces LRRK2 phosphorylation of its substrate, Rab10. Pharmacological inhibition of PI3K activity blocks the LRRK2 activity induced by inhibition of PIKfyve, as well as other endolysosomal damaging agents that are known to activate LRRK2, suggesting that PI3K activity is required for LRRK2 activation. LRRK2 also colocalizes, somewhat, with PI3P at LAMP1 positive membranes upon inhibition of PIKfyve and may interact with PI3P, as well as other PIs, through a potential interaction at its N-terminal region. The authors also find that CASM and Rab12/29 are required for LRRK2 activation by inhibition of PIKfyve, consistent with prior reports using STING activation or lysosomal damaging agents to induce LRRK2 activity. These results lead the authors to propose that PI3P, CASM, and Rab12/29 are required upstream of LRRK2 activation - either simultaneously and/or separately (it is not clear). The strengths of the manuscript are that a variety of approaches, including biochemical analysis of bulk lipids, imaging, and in vitro analysis, are used to interrogate how PI3K activity may be contributing to the activation of LRRK2. However, the overall analysis using these approaches is rather preliminary in nature and lacks proper experimental controls, which makes it difficult to make clear conclusions about the findings. Further, the quality of the imaging data is far below the standards of this journal and needs to be improved so that proper conclusions can be made regarding LRRK2 localization, which is an important component of the manuscript. While the results are interesting and would address important questions regarding the role of PIPs in LRRK2 localization and activation at endolysosomal membranes, substantial work is necessary to more rigorously and comprehensively address the questions posed and provide clear mechanistic insight.

Major points

1. The main claim of the paper that the PIKfyve inhibitor induces LRRK2 activation by "increased PI3P levels" needs much more rigorous experimental analysis to more directly test the role of PI3P/PI3K, and rule out the role of PI35P2, in LRRK2 activation.

The authors primarily use pharmacological inhibition of PI3K to support their claims that PI3P is required upstream. Orthogonal approaches to modulate PI3K activity (ideally both increasing and decreasing activity for proper controls), that are not pharmacological, are necessary to more directly test the role of PI3K/PI3P on LRRK2 activation in cells and strengthen the inhibitor data.

It appears the authors may try to do this with the use of Tat-D11, but can the authors provide evidence or a reference that Tat-D11 increases PI3K activity and its specificity? (there are no references in this section of the text) And also test whether PI3P is required for the Tat-D11 induced LRRK2 kinase activity with the VPS34 inhibitor? Specificity of this agent for PI3K activity is unclear and the potential for other cellular effects related to or downstream of the reported autophagy effect makes the relevance of these experiments for the PI3P mediated LRRK2 activation questionable.

Further, Apilimod is a PIKfyve inhibitor, yet the role of PI35P2 in LRRK2 activation is not adequately addressed. The authors do nicely detect bulk phosphoinositide levels by mass spec and do not see robust changes in PI35P2 levels with apilimod or addition of the VPS34 inhibitor. These results may contribute to the conclusion that it must be the increase in PI3P that positively regulates LRRK2 kinase activity, rather than PI35P2 playing some inhibitory role. However, PI35P2 is a low abundance, highly localized lipid (https://doi.org/10.1042/BST20150174), and small, local changes may not be detected by such bulk whole-cell population analysis. Additional experiments are necessary to convincingly rule out the effect of reducing PI35P2 levels in the activation of LRRK2.

Testing whether other PIPs are contributing to LRRK2 activation would serve as useful negative controls, especially since the authors show that LRRK2 may also interact with other PIPs in vitro in Fig. 6 and PIKfyve has been reported to modulate PI4P distribution (DOI: 10.1038/s41467-025-65798-6)..

2. The mechanistic relationship between PI3P, Rab12/29, and CASM in LRRK2 activation is unclear and confusing. All appear to be required for activating LRRK2, to some extent, with a variety of stimuli, but their relationship and order of operations for activating LRRK2 with Apilimod treatment is not clear - are all required simultaneously at the same membranes to recruit and activate LRRK2 with the loss of any one disrupting LRRK2 phosphorylation of Rab10, are these separate events recruiting and activating LRRK2 at different membranes, or are they linearly related upstream/downstream of each other? The authors are not very clear on their relationship, stating at one point that "each component separately participates in LRRK2 activation, forming a rather complicated LRRK2 activation mechanism" (lines 333-334), and then "Therefore, simultaneous binding of LRRK2 to such membranes as well as Rab and CASM components may ensure the specificity of its localization." (line 344). The finding that PI3P is involved in LRRK2 activation, along with confirming prior evidence that Rab12/29 and CASM are also involved in this distinct stimulatory context, is interesting, but all of that is left rather open-ended, making it difficult to derive a clear model. If the mechanisms here can be further worked out, it would substantially improve the manuscript and advance the field.

Further, throughout the manuscript the authors state that LRRK2 activation by apilimod is due to "increased PI3P levels" - this is confusing as the authors themselves are showing evidence that the mechanism of LRRK2 activation by apilimod is more complex, requiring also Rab12/29 and CASM, and not just an increase in PI3P levels. Please consider revising this language in the text.

3. The relationship between PIKfyve inhibition and CASM is unclear. Does apilimod treatment actually induce CASM? Determining whether the LC3B and GABARAP lipidation occurs and whether that lipidation is driven by CASM would provide some clarity in the relationship of CASM to LRRK2 activation with PIKfyve inhibition. Probing for LC3B/GABARAP lipidation in the knockdown and CASM-deficient experiments in Fig. 3 would also provide necessary controls to demonstrate efficiency of knockdown on lipidation. Further, a recent preprint reports opposing results (doi: https://doi.org/10.1101/2025.11.19.689251), showing that ATG16L1 and CASM (from KO and SopF expression) are not required for Apilimod induced LRRK2 activation - can the authors address this discrepancy?

4. While visualizing LRRK2 localization relative to PI3P and endolysosomal membranes in Fig. 5 is an important component of the strengths of the manuscript, the imaging quality needs to be drastically improved to be able to properly interpret these experiments. For example, LRRK2 localization with the PI3P sensor in panel A is not clear, and thus the colocalization is not convincing. In panels B and E, LRRK2 localization with the PI3P sensor is somewhat more apparent, however there is clearly LRRK2 accumulation that is not co-localized with the PI3P sensor, suggesting other mechanisms for LRRK2 recruitment that may not be PI3P-dependent, which needs to be acknowledged (and may inform questions above in points 2 and 3). Panel E needs an untreated control and quantification of co-localization with LAMP1 as the LAMP1 signal looks abnormally cytoplasmic (maybe background), and it is not clear whether the accumulation of LRRK2 signal completely colocalizes. The authors note challenges in imaging and may refer to https://doi.org/10.1083/jcb.202408158 and https://doi.org/10.1083/jcb.202508058 for more commonly used PI3P reporter constructs (just a note). Lastly, the authors have previously reported that LRRK2 is not recruited to lysosomes upon Vacuolin-1 treatment (Eguchi et al., 2018), which is another PIKfyve inhibitor. Can the authors address this discrepancy with their prior publication?

5. In Fig. 6C, the LRRK2 band in the PentaA mutant appears to be much less than the WT and other mutants, thus it is not clear whether the observed reduction in pRab10 is due to the mutations or due to less or unstable expression of LRRK2. This undermines the interpretation that the mutations "significantly suppressed LRRK2 activation" and requires further experimental interrogation.

Minor points

1. In the paragraph describing LAP and Nox2 related CASM (Lines 218-230), the authors acknowledge that LAP is a distinct type of CASM that requires PI3P and Nox2, while other forms of CASM do not; however, throughout the paragraph they interpret the Nox2-related results as just "CASM". This made it a bit confusing to parse the relationship of the results to "LAP-related CASM" vs the common CASM pathway. I know the nomenclature of this field is confusing in general, but for interpretation of the results and for a general audience it would help to be specific here that this is "LAP-related" CASM or something of the sort.

2. Please check citations - there are many statements made without in-text references.

3. Many sentences are written a bit confusingly. Additional copy and line editing would improve readability.

4. Please check the statistics in Figure 5C-D and provide more information for how the analysis was done - how many cells, across how many experiments, etc. The change from 0.2 to ~0.3 PCC seems rather small for statistical significance, especially with such large variance. Please also be consistent with the Y axis ranges in the plot.

Author response to reviewers' comments

We sincerely thank all three reviewers for their thorough review of our paper and insightful comments. Although we received various strict critiques, the editor thoughtfully granted us another opportunity to revise this paper, so we carefully reviewed all concerns raised by the reviewers and made necessary revisions. We believe that these revisions, while not necessarily perfect, address most of the reviewers' concerns and greatly improve the clarity and quality of the manuscript.

Below, we provide a point-by-point response to each comment and describe the changes made in the revised manuscript, which are also highlighted in yellow in the main text. We have also attached Source Data files consisting of fully uncropped immunoblot images.

Reviewer #1

In this paper, Kuwahara et al. explore the role of phosphoinositides in LRRK2 kinase activation. There are currently 3 preprints out (including this submission, one other in review at this journal), showing that PI3,5 "PIKfyve" kinase inhibition causes lysosome swelling and LRRK2 activation. This team was the first to show previously that ionophores such as chloroquine and the peptide LLOME activate LRRK2. This reviewer very much appreciates the high quality of the data herein-the gels are very beautiful and carefully generated. There are also interesting results presented but the conclusions are not yet supported by the data and some of the findings are perplexing and don't quite make sense at this stage.

>> Thank you very much for your valuable feedback. We have revised our paper to clarify the perplexing points as much as possible.

1. The authors would like to conclude the PI3P activates LRRK2. Yet in figure 1C and 2B-E, in the control reactions, PI3 kinase inhibitors (depleting PI3P) activate LRRK2. So the authors cannot state that PI3P activates LRRK2 under control conditions because they show it does not.

>> This point is absolutely correct. It was a leap to imply that PI3P changes could account for even smaller alterations in low LRRK2 activity under steady-state conditions. We should have stated that an increase in PI3P is involved when LRRK2 is hyperactivated. Therefore, we have added the following description in **lines 138-141**: "*Treatment with class III PI3K inhibitors alone did not further reduce the low level of Rab10 phosphorylation but rather tended to increase it (Fig. 1C-F), implying that low LRRK2 activity under steady-state conditions is also influenced by factors other than PI3K.*" We have now chosen to use the word "hyperactivation" of LRRK2, indicating its stronger activation under PIKfyve inhibition or CASM induction, throughout the manuscript. Title of this paper has also been changed accordingly.

As this reviewer pointed out, steady-state LRRK2 activity increases slightly under VPS34-IN1 treatment. However, the increase under VPS34-IN1 treatment was not statistically significant when compared with the massive increase under PIKfyve inhibition (Fig. 1D). Also, the increase was

even smaller under treatment with another PI3K inhibitor Compound 19 (Fig. 1F), and the same was true for another inhibitor 3-MA we added this time (Fig. S1E). Since LRRK2 exerts its activity by binding to cellular membranes and LRRK2 can bind other PIPs to some extent (Fig. 6A), we consider it reasonable that LRRK2 activity still changes even under PI3P-depleted conditions.

Apilomod activates LRRK2 and causes lysosome swelling but less so in the joint presence of a PI3 kinase inhibitor.
>> We believe this is not a contradiction; PI3P levels are thought to influence both lysosomal swelling and LRRK2 activity.

In LAP type CASM, a UVRAG-Rubicon-VPS34 complex generates PI3P on phagosomes to support LC3 lipidation, so VPS34 is functionally important. In other CASM contexts, ATG16L1 is recruited via V ATPase and membrane stress, allowing CASM to proceed largely independently of VPS34. Perhaps STING activation goes through the latter route while swollen lysosomes use the former? Specific requirements for components of the various pathways should be tested for their possible roles.
>> As this reviewer pointed out, LAP-type CASM involves an increase in phagosomal PI3P mediated by VPS34, while the increased PI3P causes LAP through the activation of NOX2, so this study has examined the involvement of NOX2. As shown in Fig 3H-I, NOX2 inhibitor treatment did not suppress LRRK2 hyperactivation upon PIKfyve inhibition, suggesting that LAP is not involved in this process. Since other CASM types do not require VPS34/PI3K activity (PMID: 25484071, 29317426), we concluded that direct activation of LRRK2 by PI3P occurs in parallel with CASM.

As for whether swollen lysosomes involve the “former” or “latter” CASM, Oliver Florey and colleagues have reported that the latter (i.e., CASM that is not LAP) is involved (the aforementioned paper, PMID: 25484071). They showed that an increase in osmotic pressure in swollen lysosomes triggers CASM via the V-ATPase-ATG16L1 axis.

2. In figure 2H, the authors measure lipid levels with various inhibitors. Apilimod blocks PI3,5P generation but no change is seen in this lipid in panel H? Why? This figure is comparing apilimod with chloroquine, monensin and nigericin which likely have very different mechanisms of LRRK2 activation. What about in a Clc7 KO cell line? [The authors should mention at least one preprint that has been out there on the same topic.]
>> The level of PI(3,5)P_2 is not solely determined by PIKfyve but also by lipid phosphatases such as MTMRs and Fig that metabolize PI(3,5)P_2 to PI5P or PI3P. Thus, it should not be clear whether PIKfyve inhibition actually reduces cellular PI(3,5)P_2 levels, yet many prior studies have been conducted on the assumption that PI(3,5)P_2 does decrease. This is partly because no established methods existed that can directly measure the levels of PI3P and PI(3,5)P_2 while distinguishing them from PI monophosphates (PI4P, PI5P) and diphosphates (PI(3,4)P_2, PI(4,5)P_2), which have

the same mass (regioisomers). The quantification of PIPs shown in Fig. 2H employs a method first reported in 2022 for quantifying PIPs while distinguishing between their regioisomers (Morioka et al, *Nat Commun* 2022, cited in this paper). This revealed that PIKfyve inhibition does not result in a decrease in $PI(3,5)P_2$ but rather leads to an increase in PI3P. This is a critical point but was not explained in detail, so we have added the descriptions to the result section **(page 8, 2nd paragraph)**.

The reason we compared the effects of apilimod and chloroquine in a same graph is that they share the same control sample and that we performed these quantifications simultaneously. While it is possible to present the data in two separate graphs, we decided to combine them as this makes it easier to understand their common effect of increasing PI3P levels.

Regarding CLC-7 reported in Clegg et al, *bioRxiv* 2025, we could not immediately obtain CLC-7 KO cells, so we conducted knockdown experiments by using four different siRNAs. However, as shown below, none of these siRNAs for CLC-7 did not reduce the protein expression of CLC-7, which was detected by two different antibodies. We were not sure whether this is an issue with the siRNAs or with the antibodies. However, since CLC-7 regulates lysosomal osmotic pressure that controls CASM, demonstrating the involvement of CLC-7 in this experiment will merely support the involvement of CASM. Furthermore, even if CLC-7 were involved, that would not support a decrease in $PI(3,5)P_2$, as the prior study (Leray et al, eLife 2022; PMID: 35670560) did not directly show its decrease in cells when CLC-7 is activated (but merely showed that $PI(3,5)P_2$ inhibits CLC-7 activity in vitro). Therefore, we decided not to pursue further the involvement of CLC-7, and we would appreciate the understanding. The above preprint and another one (Malankhanova et al, *bioRxiv* 2025), both reporting similar PIKfyve inhibition effect on LRRK2 activation, are now mentioned in the discussion section **(page 16, 3rd paragraph)**.

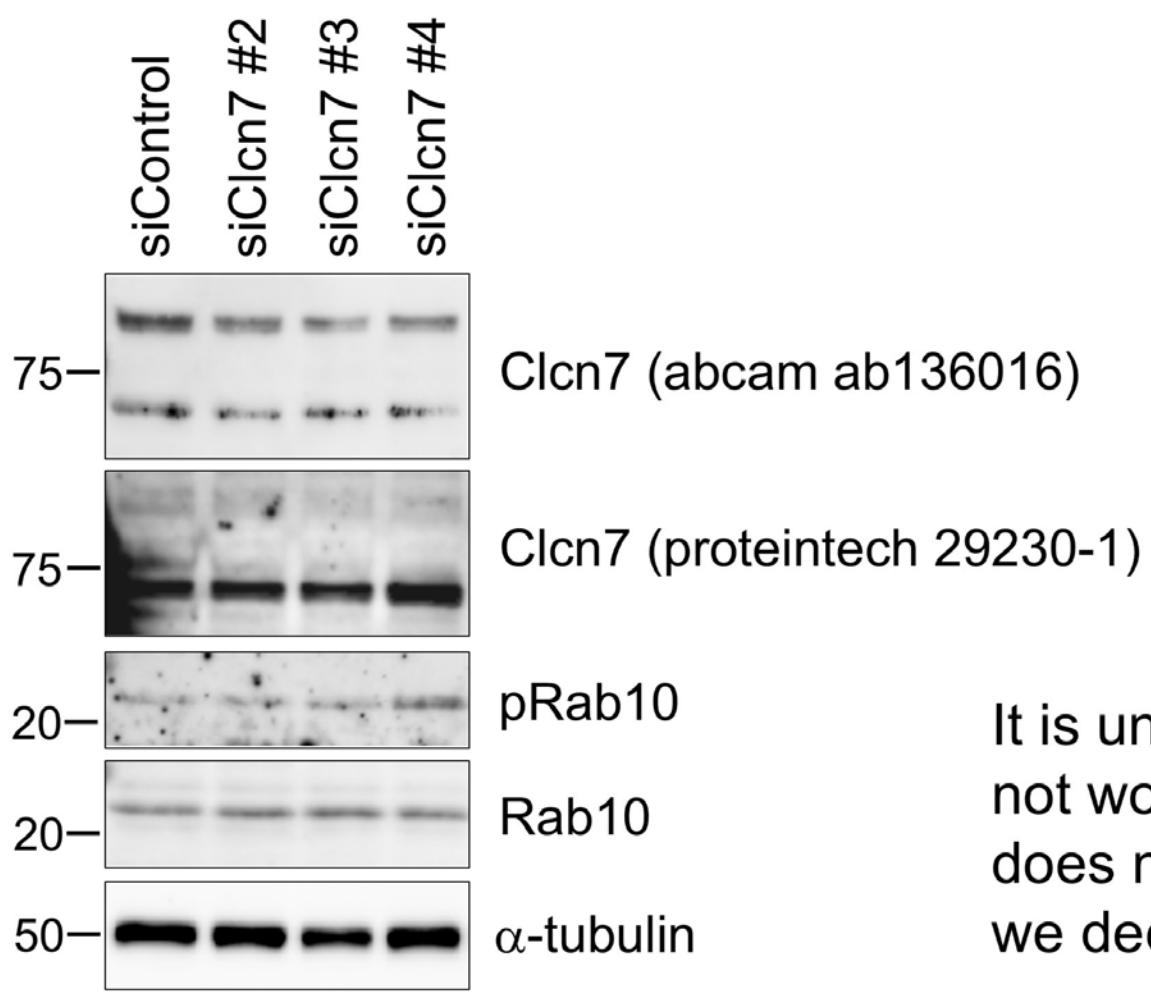

- siClcn7 #1 caused cell death
- Clcn7 (=CLC-7) band is estimated to appear around 80 kDa
- Abcam Clcn7 antibody has been used in multiple papers.

It is unclear whether siRNAs or antibodies are not working. Since the involvement of CLC-7 does not affect the conclusions of this study, we decided not to pursue this further.

3. Page 7. The authors suggest that STRESS-induced LRRK2 activation requires PI3P but the purpose of this lipid is not yet clear. Line 189: LRRK2 activity is positively correlated with PI3P-NOT

SO in control reactions. Line 215: CASM is required for activation of LRRK2 induced by increased PI3P (not shown-perhaps: "that occurs coincident with increased PI3P").

>> Lysosome stress blocks the maturation of endolysosomes, so such stress is thought to cause an increase in PI3P that are abundant in the early and immature endocytic compartment. The primary "purpose" of this increase in PI3P is likely something else, but we believe it also contributes to the activation of LRRK2 as another rational mechanism. To make this point clearer in the text, we have added the following statement in the discussion section (page 15, 2nd paragraph) "*While PI3P increase associated with endolysosomal de-maturation likely has other primary purposes, it also contributes to LRRK2 activation possibly as another rational mechanism.*".

Line 189: as noted above, "correlation" is incorrect under control conditions, so we have revised as follows (now in **lines 209-210**): "*These data strengthen the notion that hyperactivation of LRRK2 involves an increase in intracellular PI3P levels.*".

Line 215: The requirement of CASM in LRRK2 activation under PIKfyve inhibition is shown in Fig 3B-G, especially in Fig 3F-G.

4. Line 265. Not clear if LAMP staining surrounds the structures as the cells are full of lysosomes

>> We understand this point; the cells contain full of lysosomes under PIKfyve inhibition conditions. This time, we quantified the colocalization of PI3P (p40PX-EGFP) and LAMP1 using Manders' colocalization coefficient and demonstrated that colocalization increases under PIKfyve inhibition conditions **(new Fig. 5E)**. However, since these lysosomes are not fully maturated even though they are LAMP1-positive, the aim here is not to show LAMP1 positivity but simply to show the presence of PI3P/LRRK2 on large vacuoles. This is why we did not show quantitative analysis of colocalization with LAMP1 in our initial manuscript.

5. The authors would like to test if LRRK2 binds PI3P. Dot blots are not reliable or sufficient to support this conclusion-this would require flotation of LRRK2 (and mutants) with liposomes of specific phosphoinositide composition. Line 312: not yet demonstrated.

>> We agree, so we have examined the binding of recombinant LRRK2 with liposomes containing PI, PI3P or PI(3,5)P_2. We used liposomes that were both generated in house or purchased from Echelon, but the latter yielded more stable results. First, we conducted a liposome co-sedimentation assay where liposome-binding proteins are sedimented by centrifugation, but we found that LRRK2 sedimented even under mild centrifugation conditions in the absence of liposomes, possibly due to its large molecular size. As the liposomes purchased were conjugated with biotin, we next tried another pulldown assay where liposomes are collected by streptavidin magnetic beads. As a result, in the TBS buffer containing a weak detergent (NP-40 at 0.05% (v/v)), recombinant LRRK2 was pulled down with liposomes containing PI3P, and, to a slightly lesser extent, with those containing PI(3,5)P_2 (new Fig. 6B-C). Although the difference between PI3P and PI(3,5)P_2 in this assay was not statistically significant and this did not quite meet our expectations,

we thought that this is a limitation of *in vitro* experiments. Nonetheless, we were able to show the binding of LRRK2 with PI3P-containing liposomes, so we have included this data in **Fig. 6B-C**.

Line 312: if we were correct, this sentence was the following in the initial manuscript: "Lastly, we examined the possibility that LRRK2 behaves as a PI3P-dependent kinase in vitro,". These in vitro kinase assays are shown in Fig. S5A-B (which was Fig. S2A-B in the initial manuscript).

In summary, this manuscript presents a great deal of work and carefully executed pharmacological experiments. For the story to be suitable for further consideration at this journal, the authors would need to teach us what PI3P is actually doing (or bring us closer to a pathway) to give apparent LRRK2 activation upon lysosome swelling. To this reader, it is not yet clear.

We would like to express our gratitude once again for this thorough review of our manuscript. To simply state what PI3P does, our thought is that PI3P acts together with CASM components and Rab12/29 to behave as essential factors for the hyperactivation of LRRK2 and plays a role in more stably anchoring activated LRRK2 to the endolysosomal membranes. As we have reported previously (PMID: 30209220, 32919031, 38227290), LRRK2 act to facilitate lysosomal stress responses (*e.g.*, lysosomal secretion and morphology regulation), and such stressed lysosomes exhibit de-maturation and PI3P increase, so the anchoring of LRRK2 by PI3P may be a rational mechanism to facilitate LRRK2-mediated stress responses. This notion was described in more detail in the discussion section, as follows **(page 15, 2nd paragraph)**: "*The endolysosomal membrane is rich in PI3P and PI(3,5)P_2, with the ratio of PI(3,5)P_2 increasing during maturation, while stress is thought to cause de-maturation to increase PI3P levels. Therefore, simultaneous binding of LRRK2 to such membranes as well as Rab and CASM components may ensure the specificity of its localization. While PI3P increase associated with endolysosomal de-maturation likely has other primary purposes, it also contributes to LRRK2 activation possibly as another rational mechanism. Once LRRK2 is properly tethered on the membrane with the aid of PI3P, CASM components and Rab, it becomes fully stabilized and activated, boosting the lysosomal stress response via Rab phosphorylation.*".

<u>Reviewer #2</u>

Kuwahara and colleagues add new insights around the mechanisms by which lysosomal membrane damage activate LRRK2 by demonstrating that PI3P is a key requirement for LRRK2 activation following endolysosomal membrane permeabilization. Using a combination of pharmacological manipulations, they show that compounds that lead to increased PI3P levels (e.g. PIKfyve inhibition) lead to increased LRRK2 activity and suggest that this occurs in a CASM and Rab dependent manner. Overall, this work builds on previous work to increase our understanding of the signals that lead to LRRK2 recruitment to damaged endolysosomes.

While this work provides additional mechanistic clarity as to how LRRK2 activity is regulated in

response to lysosomal stress, additional work is needed to further strengthen and clarify the authors' claims to warrant publication in this journal.

>> Thank you very much for understanding the significance of our study. We have addressed the concerns as best we could, as described below.

1) LRRK2 activity is increased upon pharmacological manipulations that lead to increased PI3P levels:

a. The authors employ multiple pharmacological conditions (PIKfyve inhibition, inhibition of PI3K with multiple compounds) and multiple cell types (RAW macrophages, A549 cells, and HEK293 cells overexpression LRRK2) to demonstrate that conditions that increase PI3P levels lead to increased phosphorylation of Rab10. This data is very compelling.

b. However, additional work is needed to confirm that the increase in Rab10 phosphorylation is LRRK2 kinase dependent. Specifically, additional studies should be done demonstrating that LRRK2 kinase inhibition (e.g. with MLi-2) reduced Rab10 phosphorylation in response to modulating PI3P levels. They include this control in their studies using the TAT-D11 peptide but it is not included in any of their pharmacological studies in Figures 1C-J, and this data is required to be confident that this truly leads to an increase in LRRK2 kinase activity.

>> We are sorry for not showing basic data on Rab10 phosphorylation. This phosphorylation at Thr73 has been well established as dependent solely on LRRK2 kinase activity, but we have now demonstrated in **Fig. S1A** that Rab10 phosphorylation upon PIKfyve inhibition is fully suppressed by the LRRK2 inhibitor MLi-2. Additionally, the data showing the combined treatment with Tat-D11 and MLi-2 were retained but moved to Fig. S1F-H due to the addition of new data on Tat-D11 (Fig. 1L-M).

c. All of the studies in Figure 1 rely on pharmacological methods for increased PI3P levels. The use of orthogonal genetic approaches to modulate PI3P levels (e.g. PIKfyve KD/KO) would also be helpful supportive evidence of their conclusions.

>> We performed knockdown of Pikfyve using two different siRNAs. As shown in new **Fig. S1B-C**, treatment with either siRNA for 48 hours significantly reduced PIKfyve expression and increased Rab10 phosphorylation. Expression levels of LRRK2 and Rab10 were not altered. These data support the results of pharmacological experiments.

d. Additionally, the authors suggest that the effects reported by many in the field using compounds that damage lysosomes or perturb lysosomal homeostasis more generally lead to LRRK2 activation in a manner dependent on PI3P. These conclusions would also be further strengthened if shown using an orthogonal genetic approach in addition to treatment with VPS34-IN1 or Cmpd19 to reduced PI3P levels.

>> This point was also raised by Reviewer#3, so we performed knockdown of Vps34 that comprises

the core of PI3K. We treated cells with four different Vps34 siRNAs (from Dharmacon) for 48 hours, but unfortunately, every siRNA treatment resulted in either complete cell death or a drastic reduction in cell numbers, making it impossible to proceed with the analysis. PI3K is considered essential for cell survival and proliferation, and its inhibition for ~48 hours likely caused a significant effect. On the other hand, pharmacological inhibition lasts only 2-3 hours, which might have minimal effects on other cell functions.

That said, since we felt that using just two types of PI3K inhibitors was still insufficient, we additionally tested the effects of 3-methyladenine (3-MA), another classical PI3K inhibitor. As shown in new **Fig. S1D-E**, 3-MA treatment strongly suppressed the increase in Rab10 phosphorylation induced by PIKfyve inhibitors, similar to VPS34-IN1 and Compound 19. Therefore, we concluded that the suppression of Rab10 phosphorylation is, in fact, an effect of PI3K inhibition.

2) PI3P-mediated activation of LRRK2 requires CASM and specific Rab 12 and 29:

a. The authors show that Rab10 phosphorylation induced by PIKfyve inhibition requires key factors involved in CASM using KD of ATG16L1 and ATG5 and BMDMs from mice that express mutant Atg16l1 that lacks the WD40 domain due to a stop codon inserted after E230. This suggests that PI3P-dependent regulation of LRRK2 activity still requires the CASM machinery. They also rule out that the PI3P-Nox2 pathway upstream of CASM does not mediate LRRK2-dependent activation via PIKfyve inhibition.

b. These data overall strongly support their conclusions that PI3P-mediated activation of LRRK2 depends on CASM. In Figure 3, the genetic manipulations to inhibit CASM lead to reduced but not absent increases in pRab10 levels (the Atg5 and ATG16l1 and E230 conditions reduced pRab10 levels following apilimod treatment but do not bring it back to baseline levels). Do the authors think that additional non-CASM dependent mechanisms for inducing LRRK2 activation following apilimod treatment are occurring? Or is this due to residual CASM activity (although harder to justify in the BMDM model)? It would be helpful to add additional text or data to explain this discrepancy.

>> We do not believe that LRRK2 activity is 100% regulated by CASM. As shown in this paper, LRRK2 activation requires not only CASM components but also PI3P and Rab12/29. These factors can simultaneously bind to the different sites in the N-terminal region of LRRK2, inducing full activation of LRRK2 (which is now stated in the last sentence of the result section, page 14). Thus, although disruption of any single factor reduces LRRK2 activation and membrane binding, LRRK2 is thought to still retain some activity as long as other factors are present. This point is now described in the discussion section **(page 15, 3rd paragraph)** as follows: "*Our data suggested that PI3P-rich membranes, CASM components and Rab12/Rab29 all act as essential factors for LRRK2 activation. Although their detailed relationship remains debatable, these factors are thought to act both in parallel and in tandem. First, they can simultaneously bind to different sites in the N-terminal region of LRRK2, enabling their parallel action to fully stabilize LRRK2 on membranes and activate it. Thus, depletion of any single factor significantly reduced LRRK2 activation, whereas it did not*

completely cancel LRRK2 activation in the presence of other factors.".

c. For their experiments assessing Rab12 and 29 knockdown (Figure 4), there is no baseline pRab10 levels in control RAW macrophage cells in the blots shown. Is this due to an exposure issue or due to no detection in this experiment? This has impacts on the quantification in terms of fold change shown in C and D and confidence in effect sizes observed.

>> The baseline bands for phospho-Rab10 are visible in the control samples, albeit faint, and quantification was possible. Attached below are the uncropped immunoblot pictures with the signal intensity increased. Note that the six bands visible on the left and right in each picture represent two biological replicates.

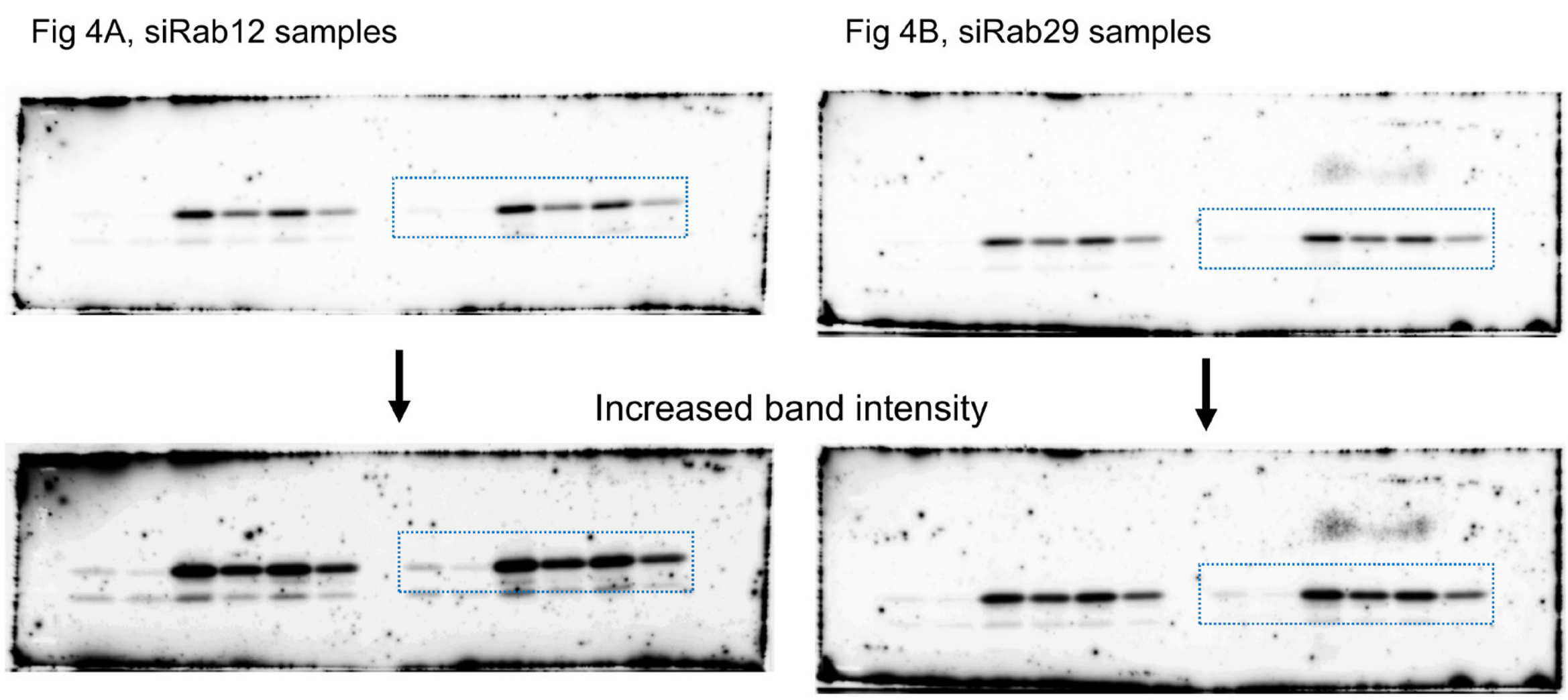

3) LRRK2 and pRab10 levels colocalize with PI3P on lysosomes:

a. The authors perform microscopy-based studies to establish that PI3P (visualized using a genetically-encoded probe) and LRRK2 or pRab10 colocalize after treatment with apilimod, YM201535 or CQ. They show that there is increased colocalization with the PI3P-detecting probe and LRRK2/pRab10 following treatment with agents that lead to increased PI3P levels.

b. They visualize endogenous LRRK2 using an antibody; however, imaging-based assessment of endogenous LRRK2 is notoriously difficult given the available antibodies. To add confidence to this data, they should confirm that this antibody is LRRK2 selective through comparison to signal seen in LRRK2 KO or LRRK2 siRNA-treated RAW264.7 cells.

>> The specificity of the immunostaining using the LRRK2 antibody (MJFF2) shown in Fig. 4 was previously confirmed in our paper (Eguchi et al, *PNAS* 2018, Fig. S2B, PMID: 30209220) using *Lrrk2* KO cells. However, we have not shown this specificity in this paper, especially under PIKfyve inhibition conditions, so we performed immunostaining of LRRK2 knockout RAW264.7 cells treated with apilimod, YM2013636 or chloroquine using the MJFF2 antibody. As shown in new **Fig. S3**, endogenous LRRK2 was not stained in any of the KO cells examined.

Of note, we sometimes hear from other LRRK2 researchers that the MJFF2 antibody cannot

detect endogenous LRRK2 by immunocytochemistry. Our immunostaining protocol involves overnight immersion in 100% ethanol after PFA fixation, which we confirmed to be a critical step. This methodology is described in the method section (page 22), as in our previous papers.

c. Additionally, they show images of colocalization between LAMP1, LRRK2, and their PI3P probe but do not provide any quantification as to whether this is increased following apilimod or CQ treatment. No untreated images are shown in panel 4E, which also makes it difficult to understand whether this is an increase observed following treatments that elevate PI3P levels. Additional controls should be included to show the staining pattern of these under baseline conditions and to quantify the colocalization following treatment.

>> The lack of quantitative data and untreated images in original Fig. 5E was also pointed out by Reviewer#3, so we provide responses to these issues as follows (which are similar to those to Reviewer #3). First, regarding the lack of untreated images, we presented new **Fig. 5A** showing the colocalization of PI3P (p40PX-EGFP), LRRK2 and LAMP1 in four samples including controls, given the comment from Reviewer#3 that the quality of some images in original Fig. 5A was poor.

Quantifying colocalization with LAMP1 was somewhat challenging: while large LAMP1-positive vesicles are abundant in the cytoplasm upon treatment with apilimod, YM201636 or chloroquine, PI3P and LRRK2 are found only to a subset of these vesicles. Consequently, analysis using Pearson's coefficient indicated that there was little colocalization (see Figure below, left). Then, we adopted Manders' colocalization coefficient—a simpler assessment method—to determine the proportion of LAMP1-positive areas within PI3P-positive areas, and found that colocalization was enhanced upon treatment with apilimod, YM201636 or chloroquine (see Figure below, right, which is now presented in new **Fig. 5E**). Although Manders method has some drawbacks that it considers only the area of fluorescence without accounting for its intensity, and that the results can vary depending on threshold settings, we believe it serves as an indicator of the degree of overlap between PI3P and LAMP1.

Of note, these LAMP1-positive vesicles under stress conditions are not expected to be fully maturated compared to normal lysosomes, so the aim here is not to show LAMP1 positivity but simply to show the presence of PI3P/LRRK2 on large vacuoles. This is why we did not show quantitative analysis of colocalization with LAMP1 in our initial manuscript.

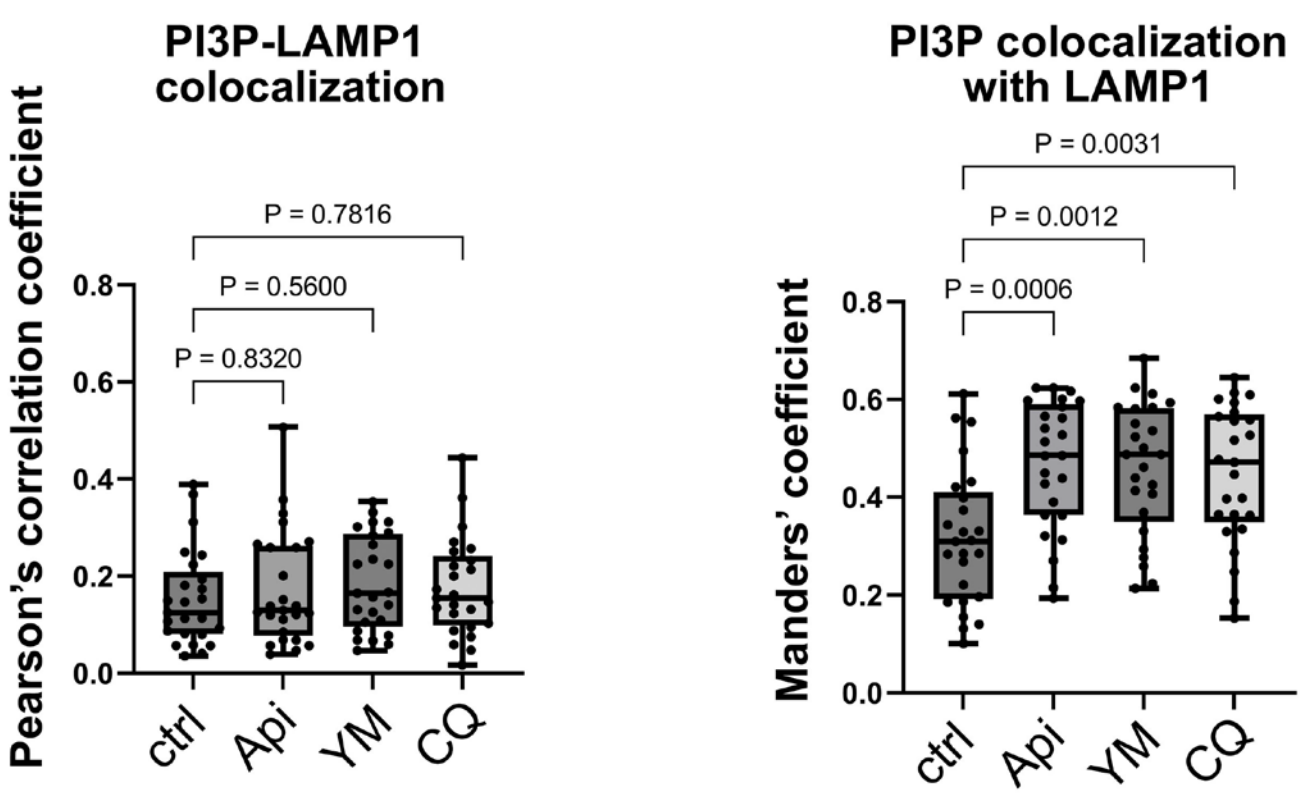

4) PI3P regulates LRRK2 activation through direct binding via N-terminal positively charged amino acids in LRRK2:

a. The authors perform elegant studies assessing whether PI3P leads to increased LRRK2 activation by directly binding to LRRK2 and identify a cluster of positively-charged residues on the N-terminus of LRRK2 that contribute to this.

b. Their data shows that the PentaA construct in which five amino acids in this region of LRRK2 are substituted to alanine leads to reduced binding of PI3P. This proposed biochemical mechanism would be further strengthened with additional data assessing the localization of the PentaA LRRK2 variant in cells. Based on their model, the prediction would be that LRRK2 localization to lysosomes following PIKfyve inhibition of the Penta LRRK2 variant would be reduced, and imaging-based experiments to directly assess this would add confidence that this interaction plays a role in this process through its effects on membrane recruitment of LRRK2.

>> Thank you for proposing an experiment to analyze PentaA localization using imaging-based methods. While this would be ideal if feasible, our RAW264.7 cells used for the localization analysis are difficult to transfect plasmids, which is now described in the main text **(page 11, line 284)**. Although p40PX-EGFP could be introduced at low frequencies by lentivirus due to its low molecular weight nature, introducing LRRK2 (2527 amino acid) is almost impossible. Furthermore, even if this is feasible, observation of LRRK2 localization on the endolysosomal membrane is only possible for endogenous protein and is difficult with overexpression. We previously confirmed that LRRK2 overexpressed in cells such as HEK293 cells localizes abundantly in the cytoplasm and on microtubules, making it difficult to observe its localization on the endolysosomal membrane. For these reasons, it was difficult to analyze the localization of PentaA LRRK2 through imaging-based observation.

5) Novelty of observations and context within existing literature:

a. The authors' data provides compelling support that PI3P is another key piece of the puzzle around how LRRK2 is activated in response to endolysosomal stress. The novelty of the work should be further articulated in the discussion, as treatment with apilimod has been previously shown to lead to LRRK2 activation. This work does significantly extent the previously published findings, but additional text referencing previous findings and articulating the novel insight from this work would be helpful in the Discussion section.

> This manuscript was posted to *bioRxiv* at roughly the same time as the other two preprints (all three within a month) and is not a follow-up to those two preprints. (Note: this manuscript was submitted to *this journal* on 12/16/2025 but it takes about one month before it was sent to the reviewers). Unlike our paper focusing on PI3P increase, the other two papers claim—based on the assumption that PIKfyve inhibition decreases $PI(3,5)P_2$ (which is not confirmed in their paper)—that PIKfyve inhibition-induced increase in osmotic pressure leads to LRRK2 activation. They also reported that

this activation is independent of CASM, while increase in osmotic pressure is already known to be induced by chloroquine or monensin (Florey et al, *Autophagy* 2015, PMID: 25484071), which are exactly the CASM-causing stimuli. Our current paper suggests that an increase in PI3P contributes to LRRK2 hyperactivation in addition to CASM, which is more novel. To explain this in detail, we have added a whole paragraph in the discussion section **(page 16, 3rd paragraph)**.

Reviewer #3

Kuwahara et al., set out to address the gap in knowledge regarding the role of lipids in LRRK2 kinase activity. The authors focus on phosphoinositides, finding that pharmacological inhibition of PIKfyve, the major kinase converting PI3P to PI35P2, induces LRRK2 phosphorylation of its substrate, Rab10. Pharmacological inhibition of PI3K activity blocks the LRRK2 activity induced by inhibition of PIKfyve, as well as other endolysosomal damaging agents that are known to activate LRRK2, suggesting that PI3K activity is required for LRRK2 activation. LRRK2 also colocalizes, somewhat, with PI3P at LAMP1 positive membranes upon inhibition of PIKfyve and may interact with PI3P, as well as other PIs, through a potential interaction at its N-terminal region. The authors also find that CASM and Rab12/29 are required for LRRK2 activation by inhibition of PIKfyve, consistent with prior reports using STING activation or lysosomal damaging agents to induce LRRK2 activity. These results lead the authors to propose that PI3P, CASM, and Rab12/29 are required upstream of LRRK2 activation - either simultaneously and/or separately (it is not clear). The strengths of the manuscript are that a variety of approaches, including biochemical analysis of bulk lipids, imaging, and in vitro analysis, are used to interrogate how PI3K activity may be contributing to the activation of LRRK2. However, the overall analysis using these approaches is rather preliminary in nature and lacks proper experimental controls, which makes it difficult to make clear conclusions about the findings. Further, the quality of the imaging data is far below the standards of this journal and needs to be improved so that proper conclusions can be made regarding LRRK2 localization, which is an important component of the manuscript. While the results are interesting and would address important questions regarding the role of PIPs in LRRK2 localization and activation at endolysosomal membranes, substantial work is necessary to more rigorously and comprehensively address the questions posed and provide clear mechanistic insight.

>> Thank you very much for the critical feedback on the strengths and weaknesses of our paper. As described below, we have made revisions to the best of our ability. Although we faced some challenges—such as the inability to prepare super-resolution images—we believe we have mostly addressed the concerns raised.

Major points

1. The main claim of the paper that the PIKfyve inhibitor induces LRRK2 activation by "increased PI3P levels" needs much more rigorous experimental analysis to more directly test the role of

PI3P/PI3K, and rule out the role of PI35P2, in LRRK2 activation.

The authors primarily use pharmacological inhibition of PI3K to support their claims that PI3P is required upstream. Orthogonal approaches to modulate PI3K activity (ideally both increasing and decreasing activity for proper controls), that are not pharmacological, are necessary to more directly test the role of PI3K/PI3P on LRRK2 activation in cells and strengthen the inhibitor data.

It appears the authors may try to do this with the use of Tat-D11, but can the authors provide evidence or a reference that Tat-D11 increases PI3K activity and its specificity? (there are no references in this section of the text). And also test whether PI3P is required for the Tat-D11 induced LRRK2 kinase activity with the VPS34 inhibitor? Specificity of this agent for PI3K activity is unclear and the potential for other cellular effects related to or downstream of the reported autophagy effect makes the relevance of these experiments for the PI3P mediated LRRK2 activation questionable.

>> First, we are sorry for not fully explaining Tat-D11 in the main text. Tat-D11 is a modified version of the peptide Tat-Beclin 1, which has been widely used as an autophagy inducer, and multiple papers have demonstrated its ability to increase PI3K activity (PMID: 30581147, 33222586, reviewed in 36172279). These references are now cited in the main text **(page 7, line 159)**. To test whether PI3P production is required for Tat-D11-induced activation of LRRK2, we treated cells with a PI3K inhibitor Compound19 in addition to Tat-D11 and found that such LRRK2 activation was canceled by Compound19. This data is shown in new **Fig. 1L-M**. Also, the previous data on the treatment with both Tat-D11 and LRRK2 inhibitor MLi-2 have been moved to **Fig. S1F-H**.

We agree that the above data is not sufficient to confirm the specific role of PI3P in LRRK2 activation, since Tat-D11/Beclin 1 also upregulate autophagy and its downstream pathways via PI3P production. However, since it is difficult to eliminate only downstream while preserving PI3P production, we suggested the role of PI3P in LRRK2 activation by combining other data in this paper. We also believe that our previous finding that CASM, but not autophagy, contributes to LRRK2 activation (Eguchi et al, *JCB* 2024, PMID: 38227290) serves as additional evidence to rule out the effect of autophagy itself.

We also agree that the lack of involvement of PI(3,5)P_2 should be shown more directly, given two other bioRxiv preprints posted around the same time as this paper. Since we also wanted to confirm the involvement of PI(3,5)P_2, we examined the quantitative changes in all eight PIPs, including PI3P an PI(3,5)P_2, by specialized mass spec that can separately measure each PIP regioisomer. As shown in Fig. 2H, PIKfyve inhibition increased PI3P levels as well as LRRK2 activity while maintaining PI(3,5)P_2 levels. Also, additional PI3K inhibition decreased PI3P levels as well as LRRK2 activity while maintaining PI(3,5)P_2 levels. Together, we believe these data provide evidence that PI3P is more directly involved in LRRK2 activity than PI(3,5)P_2.

Regarding the point that pharmacological approach is not sufficient to validate the effect of PI3K inhibition, this was also raised by Reviewer#2, so we performed knockdown of Vps34 that comprises the core of PI3K. We treated cells with four different Vps34 siRNAs (from Dharmacon) for 48 hours, but unfortunately, every siRNA treatment resulted in either complete cell death or a

drastic reduction in cell numbers, making it impossible to proceed with the analysis. PI3K is considered essential for cell survival and proliferation, and its inhibition for ~48 hours likely caused a significant effect. On the other hand, pharmacological inhibition lasts only 2-3 hours, which might have minimal effects on other cell functions.

That said, since we felt that using just two types of PI3K inhibitors was still insufficient, we additionally tested the effects of 3-methyladenine (3-MA), another classical PI3K inhibitor. As shown in new **Fig. S1D-E**, 3-MA treatment strongly suppressed the increase in Rab10 phosphorylation induced by PIKfyve inhibitors, similar to VPS34-IN1 and Compound 19. Therefore, we concluded that the suppression of Rab10 phosphorylation is, in fact, an effect of PI3K inhibition.

Further, Apilimod is a PIKfyve inhibitor, yet the role of PI35P2 in LRRK2 activation is not adequately addressed. The authors do nicely detect bulk phosphoinositide levels by mass spec and do not see robust changes in PI35P2 levels with apilimod or addition of the VPS34 inhibitor. These results may contribute to the conclusion that it must be the increase in PI3P that positively regulates LRRK2 kinase activity, rather than PI35P2 playing some inhibitory role. However, PI35P2 is a low abundance, highly localized lipid (https://doi.org/10.1042/BST20150174), and small, local changes may not be detected by such bulk whole-cell population analysis. Additional experiments are necessary to convincingly rule out the effect of reducing PI35P2 levels in the activation of LRRK2.
>> Considering the two preprints (Clegg et al, and Malankhanova et al) posted to *bioRxiv* around the same time as this paper, we fully understand the argument that PI(3,5)P_2 rather than PI3P should be focused. First, our balk quantification of PI3P and PI(3,5)P_2 in Fig 2H is considered valid, because 1) concentrations of these PIPs are as expected, with the level of PI(3,5)P_2 lower than that of PI3P (as this reviewer pointed out), and 2) the level of PI(3,5)P_2 decreased upon CQ treatment but not with apilimod, ensuring that the detection limit lies even lower. Next, regarding the local decrease in PI(3,5)P_2 levels, we cannot rule out the possibility that it occurs in addition to PI3P increase upon LRRK2 hyperactivation. However, it is somewhat unlikely that a local decrease in such a small lipid fraction alone can explain the bulk and significant activation of LRRK2, and it is difficult to demonstrate their association since no known treatment exists that reliably reduces cellular PI(3,5)P_2 levels, which are also regulated by multiple phosphatases. Rather, based on our data, we maintain that the increase in PI3P is the factor most strongly related to LRRK2 hyperactivation—not only because the change in PI3P levels is the most drastic and corresponds to LRRK2 activation, but also because LRRK2 is found on PI3P-rich membranes upon activation and binds to PI3P at specific sites.

Finally, while the other two preprints argue that an increase in lysosomal osmotic pressure leads to LRRK2 activation, whether this increase is accompanied by a decrease in PI(3,5)P_2 is not fully clarified. Rather, it is not contradictory if an increase in osmotic pressure is accompanied by an increase in PI3P levels. We have addressed this point, as well as the similarities and differences with the other two preprints, in the discussion section **(page 16, 3rd paragraph)**.

Testing whether other PIPs are contributing to LRRK2 activation would serve as useful negative controls, especially since the authors show that LRRK2 may also interact with other PIPs in vitro in Fig. 6 and PIKfyve has been reported to modulate PI4P distribution (DOI: 10.1038/s41467-025-65798-6).

>> Since the only significant change observed upon PIKfyve inhibition was an increase in PI3P levels (Fig. 2H), we believe that PI3P plays the most significant role in such conditions. However, as this reviewer pointed out, Fig. 6A shows that LRRK2 can bind to other PIPs as well, particularly PI monophosphates PI4P and PI5P, so LRRK2 could also be activated when stressed by stimuli that increase such monophosphates. This possibility was already discussed in the discussion section of the initial manuscript (**page 17-18, lines 445-452** in this revised manuscript), but we have now cited two studies reporting that treatment with LLOMe—which damages lysosomal membranes and increases PI4P levels—activates LRRK2 (PMID: 32643832, 33135724). Although we did not pursue this possibility since our RAW264.7 cell line showed little activation of LRRK2 upon LLOMe treatment, we believe it is worth investigating the involvement of PI4P in cells where such activation occurs.

Finally, the report raised by this reviewer—that PI4P is regulated by PIKfyve—is also interesting, but we think this is also largely dependent on the cell type. We assume that PI4P is more likely to change in cells that are susceptible to lysosomal membrane damage.

2. The mechanistic relationship between PI3P, Rab12/29, and CASM in LRRK2 activation is unclear and confusing. All appear to be required for activating LRRK2, to some extent, with a variety of stimuli, but their relationship and order of operations for activating LRRK2 with Apilimod treatment is not clear - are all required simultaneously at the same membranes to recruit and activate LRRK2 with the loss of any one disrupting LRRK2 phosphorylation of Rab10, are these separate events recruiting and activating LRRK2 at different membranes, or are they linearly related upstream/downstream of each other? The authors are not very clear on their relationship, stating at one point that "each component separately participates in LRRK2 activation, forming a rather complicated LRRK2 activation mechanism" (lines 333-334), and then "Therefore, simultaneous binding of LRRK2 to such membranes as well as Rab and CASM components may ensure the specificity of its localization." (line 344). The finding that PI3P is involved in LRRK2 activation, along with confirming prior evidence that Rab12/29 and CASM are also involved in this distinct stimulatory context, is interesting, but all of that is left rather open-ended, making it difficult to derive a clear model. If the mechanisms here can be further worked out, it would substantially improve the manuscript and advance the field.

>> We are sorry for the confusion on the relationship between PI3P, CASM and Rab12/29. We proposed a model where all these factors are simultaneously required for LRRK2 activation. As shown in **Fig. 6H** (Fig. 6F in initial manuscript), these factors can all bind simultaneously to different

sites in the N-terminal region of LRRK2, which enables parallel action of these factors to fully stabilize LRRK2 on membranes and activate it. This basic notion is now clearly stated in the last sentence of the result section **(page 14, lines 361-364)**. On the other hand, more precisely, the relationship between these essential activators might be more complex, given that PIKfyve inhibition caused CASM induction (Fig. 3F) and CASM in turn increased PI3P levels (Fig. 2H). Thus, not only parallel action but also tandem relationship is considered, so we have added an entire paragraph explaining this in the discussion section, as follows **(page 15-16, lines 389-399)**: "*Our data suggested that PI3P-rich membranes, CASM components and Rab12/Rab29 all act as essential factors for LRRK2 activation. Although their detailed relationship remains debatable, these factors are thought to act both in parallel and in tandem. First, they can simultaneously bind to different sites in the N-terminal region of LRRK2, enabling their parallel action to fully stabilize LRRK2 on membranes and activate it. Thus, depletion of any single factor significantly reduced LRRK2 activation, whereas it did not completely cancel LRRK2 activation in the presence of other factors. On the other hand, our data showed that PIKfyve inhibition caused CASM induction (Fig. 3F) and CASM in turn increased PI3P levels (Fig. 2H), supporting the existence of their tandem relationship. Thus, such complex interplay among these essential factors is thought to contribute to LRRK2 hyperactivation*.".

As for whether these essential factors act on the same membrane or on separate membrane, we assume the same membrane because each can bind simultaneously to LRRK2. However, to be strict, we cannot rule out the possibility of acting on a separate membrane, while demonstrating this would be extremely challenging, so we consider this to be beyond the scope of this paper.

Further, throughout the manuscript the authors state that LRRK2 activation by apilimod is due to "increased PI3P levels" - this is confusing as the authors themselves are showing evidence that the mechanism of LRRK2 activation by apilimod is more complex, requiring also Rab12/29 and CASM, and not just an increase in PI3P levels. Please consider revising this language in the text.
>> We agree, so we have revised the descriptions where the effect of PIKfyve inhibitors was stated simply as an increase in PI3P levels (*e.g.*, lines 235-236).

Along with this, Reviewer#1 pointed out that the level of PI3P and LRRK2 activity in cells are not necessarily "correlated", given the slight change in phospho-Rab10 in the absence of PIKfyve inhibitors, so we have halted the use of the wording "correlation". One important conclusion drawn from this study is that PI3P, along with CASM and Rab12/29, functions as an essential component for LRRK2 hyperactivation upon PIKfyve inhibition; to make this point clearer, we have now used the term "hyperactivation" throughout the text where appropriate (including the title and abstract).

3. The relationship between PIKfyve inhibition and CASM is unclear. Does apilimod treatment actually induce CASM? Determining whether the LC3B and GABARAP lipidation occurs and whether that lipidation is driven by CASM would provide some clarity in the relationship of CASM

to LRRK2 activation with PIKfyve inhibition. Probing for LC3B/GABARAP lipidation in the knockdown and CASM-deficient experiments in Fig. 3 would also provide necessary controls to demonstrate efficiency of knockdown on lipidation.

>> This is an important point, so we examined whether PIKfyve inhibition induces CASM. As shown in the newly added panels for LC3 immunoblots in **Fig. 3B and F**, lipidation of LC3B indeed elevated upon treatment with apilimod, not only with the positive control CQ. Importantly, this lipidation was suppressed in ATG16L1 WD40-deficient cells (from E230 mice) where CASM is specifically disabled (Fig. 6F), suggesting that PIKfyve inhibition does induce CASM, not just the blockage of autophagy flux following lysosome dysfunction.

On the other hand, this result does not affect our conclusion that PI3P contributes to LRRK2 activation in addition to CASM, because PI3K inhibitors suppress LRRK2 activation under CASM induction but not CASM itself (Fig. 2A-G), and the independency of PI3K from CASM has also been confirmed (PMID: 25484071, 29317426). Thus, we now propose a revised mechanism of LRRK2 activation, where PIKfyve inhibition causes PI3P increase and CASM induction, both of which contribute to LRRK2 hyperactivation in parallel. This is now explained in more detail in the result section **(page 10, 2nd paragraph)**.

Lipidation of GABARAP was only faintly detected upon treatment with CQ or apilimod, in our hands, and this signal disappeared upon knockdown of *Atg5* or *Atg16l1*. This picture, along with the one with the signal greatly intensified, is now included in new **Fig. S2**. Since this signal was so weak, we have not examined GABARAP lipidation in other experiments. Please note that the involvement of GABARAP in CASM has not been fully established, and our studies to date have not found solid evidence of its involvement.

Further, a recent preprint reports opposing results (doi: https://doi.org/10.1101/2025.11.19.689251), showing that ATG16L1 and CASM (from KO and SopF expression) are not required for Apilimod induced LRRK2 activation - can the authors address this discrepancy?

>> We cannot provide clear reasons for this discrepancy, but the CASM-suppressing effect of SopF overexpression should not be tested biochemically unless it is expressed in the majority of cells. These authors also presented data on GABARAP KO, but our unpublished data (in preparation) suggested that GARARAPL1 may be compensating for that knockout.

4. While visualizing LRRK2 localization relative to PI3P and endolysosomal membranes in Fig. 5 is an important component of the strengths of the manuscript, the imaging quality needs to be drastically improved to be able to properly interpret these experiments. For example, LRRK2 localization with the PI3P sensor in panel A is not clear, and thus the colocalization is not convincing. In panels B and E, LRRK2 localization with the PI3P sensor is somewhat more apparent, however there is clearly LRRK2 accumulation that is not co-localized with the PI3P sensor, suggesting other mechanisms for LRRK2 recruitment that may not be PI3P-dependent, which needs to be

acknowledged (and may inform questions above in points 2 and 3).

>> Since pictures are magnified to the single-cell level, there are inevitably limitations to the resolution. A super-resolution microscope is required to achieve even higher resolution, but our facility does not have such microscopes for routine use. Since Fig. 5A pictures were pointed out to be particularly unclear, we moved the pictures originally shown in Fig. 5E to new **Fig. 5A** and added LAMP1 pictures to part of them. Please also note that, just in case, our paper posted to *bioRxiv* contains pictures with significantly reduced resolution.

As for LRRK2 localization, LRRK2 is present also in the cytosol, which may cause it to appear as background signal. This cytosolic localization patten was already shown in our previous paper (Eguchi et al, *PNAS* 2018, PMID: 30209220), but to clarify this again under PIKfyve inhibition conditions, we performed LRRK2 immunostaining on *Lrrk2* knockout RAW264.7 cells and demonstrated the lack of staining even in the cytosol (new **Fig. S3**, and described in **pages 11-12, Lines 289-292**). Also, since LRRK2 binds not only to PI3P but also to other phospholipids to some extent (Fig. 6A), LRRK2 signal is likely detected even on PI3P-negative membranes, as this reviewer pointed out. We acknowledged the existence of a portion of LRRK2 signal that is not co-localized with PI3P in the result section, as follows **(page 12, lines 294-295)**: "*Since not all signals were well matched with p40PX-EGFP, the possibility remains that other PI3P-independent mechanisms for LRRK2 recruitment exist.*".

Panel E needs an untreated control and quantification of co-localization with LAMP1 as the LAMP1 signal looks abnormally cytoplasmic (maybe background), and it is not clear whether the accumulation of LRRK2 signal completely colocalizes. The authors note challenges in imaging and may refer to https://doi.org/10.1083/jcb.202408158 and https://doi.org/10.1083/jcb.202508058 for more commonly used PI3P reporter constructs (just a note).

>> The lack of quantitative data and untreated images in original Fig. 5E was also pointed out by Reviewer#2, so we provide responses to these issues as follows (which are similar to those to Reviewer #2). First, regarding the lack of untreated images, we presented new **Fig. 5A** showing the colocalization of PI3P (p40PX-EGFP), LRRK2 and LAMP1 in four samples including controls, given this reviewer's comment that the quality of some images in original Fig. 5A was poor.

Quantifying colocalization with LAMP1 was somewhat challenging: while large LAMP1-positive vesicles are abundant in the cytoplasm upon treatment with apilimod, YM201636 or chloroquine, PI3P and LRRK2 are found only to a subset of these vesicles. Consequently, analysis using Pearson's coefficient indicated that there was little colocalization (see Figure below, left). Then, we adopted Manders' colocalization coefficient—a simpler assessment method—to determine the proportion of LAMP1-positive areas within PI3P-positive areas, and found that colocalization was enhanced upon treatment with apilimod, YM201636 or chloroquine (see Figure below, right, which is now presented in new **Fig. 5E**). Although Manders method has some drawbacks that it considers only the area of fluorescence without accounting for its intensity, and

that the results can vary depending on threshold settings, we believe it serves as an indicator of the degree of overlap between PI3P and LAMP1.

Of note, these LAMP1-positive vesicles under stress conditions are not expected to be fully maturated compared to normal lysosomes, so the aim here is not to show LAMP1 positivity but simply to show the presence of PI3P/LRRK2 on large vacuoles. This is why we did not show quantitative analysis of colocalization with LAMP1 in our initial manuscript.

We also thank this reviewer for providing us with the information about the new PI3P reporters. It would be ideal to try these as well, but this method includes generation and purification of recombinant proteins which will take time, so we would like to use them in our future experiments.

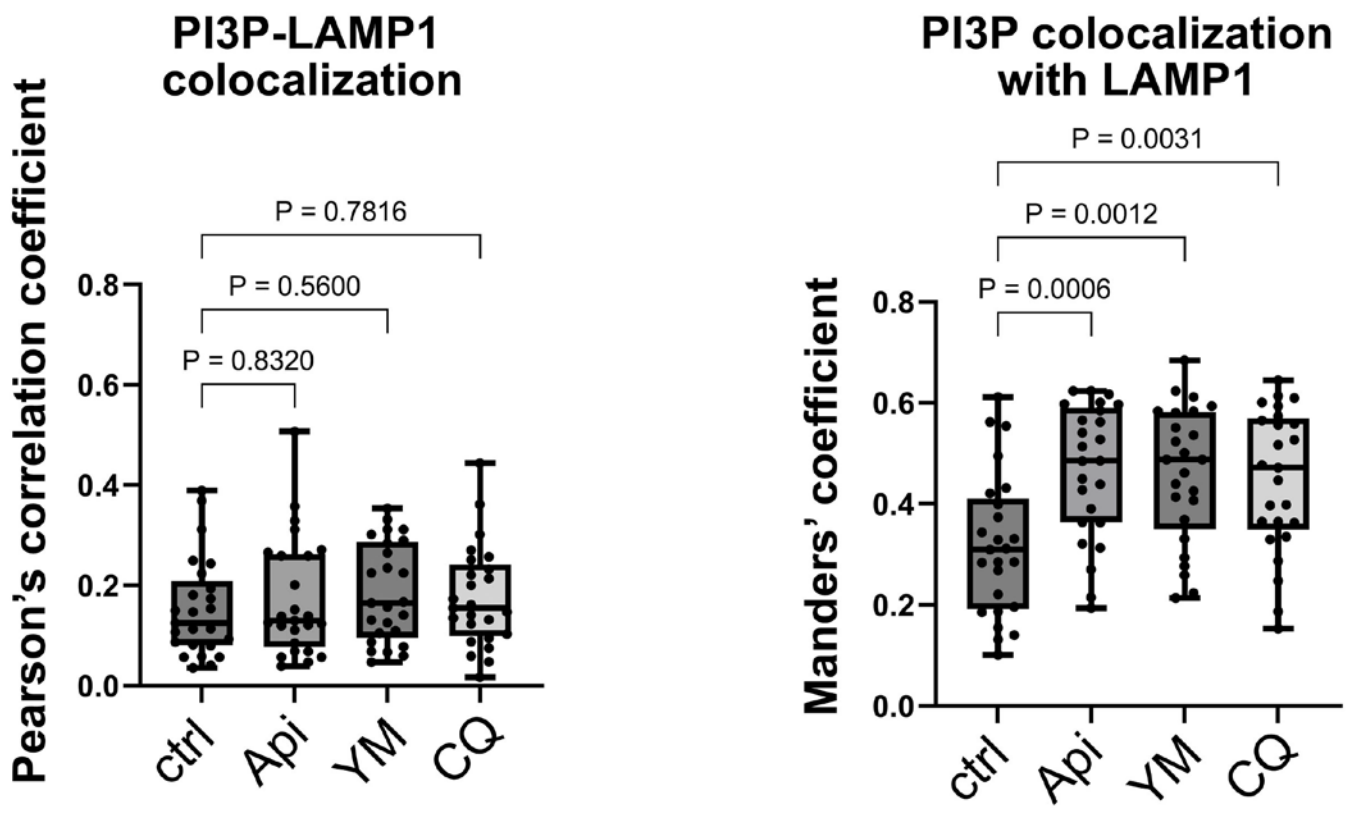

Lastly, the authors have previously reported that LRRK2 is not recruited to lysosomes upon Vacuolin-1 treatment (Eguchi et al., 2018), which is another PIKfyve inhibitor. Can the authors address this discrepancy with their prior publication?

>> We recognize this as a discrepancy. Since LRRK2 recruitment occurs less frequently with apilimod or YM201636 than with CQ, we thought we might not have noticed the recruitment when Vacuolin-1 was treated. However, when we repeated this experiment, the recruitment of LRRK2 was again hardly observed. Then, we biochemically examined the activation of LRRK2 upon Vacuolin-1 treatment and found that LRRK2 was little activated, unlike with apilimod or YM201636 (see Figure below, left). Although the exact reason of this is unclear, microscopic observation of drug-treated cells revealed that Vacuolin-1 does not appear to induce pronounced vacuole formation as compared to apilimod or YM201636 treatment (see Figure below, right), so this weaker effect might be a cause.

To further confirm that the effect of apilimod and YM201636 on LRRK2 activation is truly dependent on PIKfyve inhibition, we have now added a PIKfyve knockdown experiment using two different siRNAs. As shown in new **Fig. S1B-C**, treatment with either siRNA for 48 hours significantly reduced PIKfyve expression and increased Rab10 phosphorylation. Expression levels of LRRK2 and Rab10 were not altered. These data support the effect of apilimod and YM201636 as PIKfyve inhibitors.

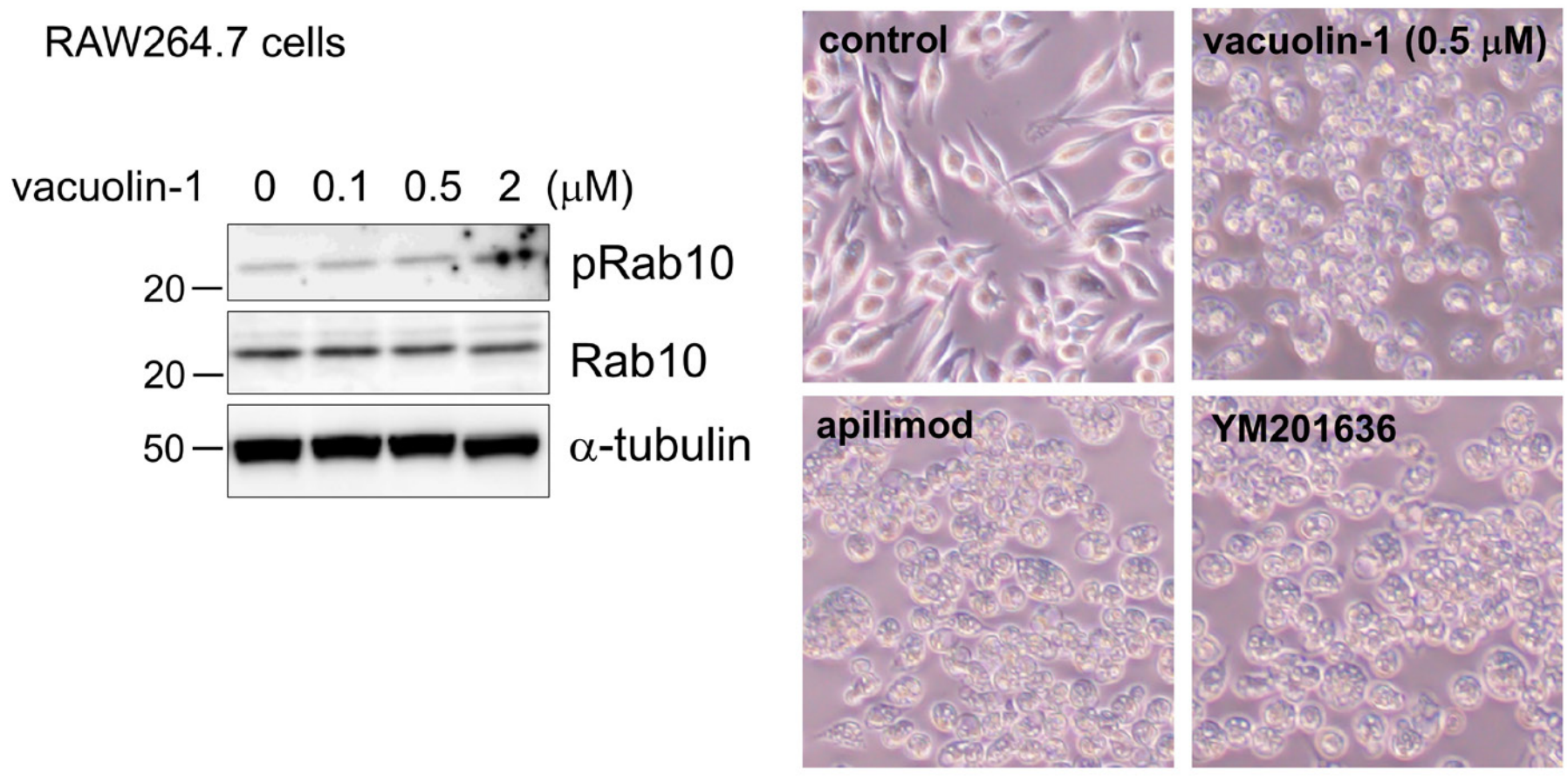

5. In Fig. 6C, the LRRK2 band in the PentaA mutant appears to be much less than the WT and other mutants, thus it is not clear whether the observed reduction in pRab10 is due to the mutations or due to less or unstable expression of LRRK2. This undermines the interpretation that the mutations "significantly suppressed LRRK2 activation" and requires further experimental interrogation.
>> This is also a valid point. The expression level of PentaA LRRK2 was actually decreased, which might be a cause of pRab10 reduction. Since N-terminal-deficient LRRK2 mutants that harbor lower membrane-binding ability are prone to become unstable, this result may still support the notion that the PentaA LRRK2 mutation reduces membrane binding. We have now added the description about this view in the result section **(page 13, lines 341-343)**.

Minor points
1. In the paragraph describing LAP and Nox2 related CASM (Lines 218-230), the authors acknowledge that LAP is a distinct type of CASM that requires PI3P and Nox2, while other forms of CASM do not; however, throughout the paragraph they interpret the Nox2-related results as just "CASM". This made it a bit confusing to parse the relationship of the results to "LAP-related CASM" vs the common CASM pathway. I know the nomenclature of this field is confusing in general, but for interpretation of the results and for a general audience it would help to be specific here that this is "LAP-related" CASM or something of the sort.
>> We agree, so in this paragraph **(page 10, 3rd paragraph)**, we have corrected all instances of "CASM"—which refers to LAP-related CASM—to simply "LAP" to avoid confusion. Also, the subtitle containing this paragraph and Fig. 3 title "PIKfyve inhibition-induced activation of LRRK2 requires CASM but not its upstream effector NOX2" were similarly confusing, and the role of CASM in LRRK2 activation is now more complex, so we have corrected them to simply "CASM is involved in LRRK2 activation upon PIKfyve inhibition".

2. Please check citations - there are many statements made without in-text references.
>> We have added citations, such as those in lines 113, 118, 159, 196-197, 203.

3. Many sentences are written a bit confusingly. Additional copy and line editing would improve readability.

>> We have made corrections wherever possible. Pease refer to the revised sentences highlighted in yellow in the text.

4. Please check the statistics in Figure 5C-D and provide more information for how the analysis was done - how many cells, across how many experiments, etc. The change from 0.2 to ~0.3 PCC seems rather small for statistical significance, especially with such large variance. Please also be consistent with the Y axis ranges in the plot.

>> We have included statistical information regarding Figures 5C-D and the newly added Figure 5E in each figure legend. As noted in the text **(page 11, lines 284-286)**, the transfection efficiency of p40PX-EGFP in our RAW264.7 cells was extremely low even with lentivirus, and since we excluded cells exhibiting high levels of expression that may compete with other proteins for PI3P binding, we analyzed a total 25 cells from the two experiments. Even with large variance, a statistically significant difference was observed. Y axis ranges in Figs 5C-E were adjusted to be consistent among them.

October 21, 2026

RE: Life Science Alliance Manuscript #LSA-2026-03939-T

Dr. Tomoki Kuwahara
The University of Tokyo
Dept. Neuropathology, Grad. Sch. Med.
7-3-1, Hongo, Bunkyo-ku
Tokyo 1130033
Japan

Dear Dr. Kuwahara,

Thank you for transferring your revised manuscript entitled "LRRK2 is hyperactivated by phosphatidylinositol 3-phosphate in conjunction with CASM" along with previous reviewer comments to Life Science Alliance. We are pleased to confirm our prior offer, also reflected in our recent correspondence. We would be happy to publish your paper in Life Science Alliance pending minor revisions as well as formatting changes necessary to meet our formatting guidelines.

Namely, a revised manuscript must include a statistical test in Figure 1D (control condition) to indicate changes induced by VPS34-IN. We further encourage you to amend your reference to Clagg et al. and we invite you to discuss the related commentary by Pfeffer et al.

MANUSCRIPT ORGANIZATION AND FORMATTING:

To avoid unnecessary delays in the acceptance and publication of your paper, please read the following information carefully. Full guidelines are available on our Instructions for Authors page, https://www.life-science-alliance.org/authors

Submission of a paper that does not conform to Life Science Alliance guidelines will delay the acceptance of your manuscript.

-Please upload all figure files individually, including the supplementary figure files; all figure legends should only appear in the main manuscript file.
-Please remove the paragraph "Online supplemental material".
-Please add an ORCID ID for the corresponding author; you should have received instructions on how to do so.
-Please add a Summary Blurb/Alternate Abstract in our system.
-Please add a Category for your manuscript in our system.
-Please add the X and Bluesky handles of your host institute/organization, as well as your own, and/or one of the authors, in our system.
-Please upload a clean manuscript file without highlights and ready for publication. The highlighted version, please upload as "Related Manuscript file".
-Please add your main and supplementary figure legends to the main manuscript text after the references section.
-Please add authors' contributions to our system as well.
-Please use the [10 author names et al.] format in your references (i.e., limit the author names to the first 10).
-Please include the full lipidomics results as a supplementary table, or provide a link to an online mass spectrometry repository in your Data Availability section.
-Please provide sequences for all primers and siRNA oligos (unless these are available from the supplier catalog numbers).
-In the methods section, please provide details on antibody concentration, illumination sources, and objectives used for imaging in the Immunocytochemistry section. Please also provide the antibody concentrations used for western blotting.

We encourage our authors to provide original source data, particularly uncropped/-processed electrophoretic blots and spreadsheets for the main figures of the manuscript. If you would like to add source data, we would welcome one PDF/Excel-file per figure for this information. These files will be linked as supplementary "Source Data" files.

It is Life Science Alliance policy that if requested, original data images must be made available to the editors. Failure to provide original images upon request will result in unavoidable delays in publication. Please ensure that you have access to all original data images prior to final submission.

If you are planning a press release on your work, please inform us immediately to allow informing our production team and scheduling a release date.

We welcome submissions of potential cover images for the issue of LSA in which your work would appear. If you have high

quality images associated with this work, please feel free to email these, with a caption, to the journal office.

LSA encourages authors to provide a 30-60 second video where the study is briefly explained. These videos will be appear embedded with the manuscript online at Life Science Alliance, and on social media to promote the published paper and authors (for examples, see https://docs.google.com/document/d/1-UWCfbE4pGcDdcgzcmiuJl2XMBJnxKYeqRvLLrLSo8s/edit?usp=sharing). Corresponding or first-authors are welcome to submit the video. Please submit only one video per manuscript. The video can be emailed to contact@life-science-alliance.org

FINAL FILES:

To upload the final version of your manuscript, please log in to your account: https://lsa.msubmit.net/cgi-bin/main.plex
You will be guided to complete the submission of your revised manuscript and to fill in all necessary information. Please get in touch in case you do not know or remember your login name.

The following items are required for acceptance.

-- An editable version of the final text (.DOC or .DOCX) is needed for copyediting (no PDFs).

-- High-resolution figure, supplementary figure and video files uploaded as individual files: See our detailed guidelines for preparing your production-ready images, https://www.life-science-alliance.org/authors

-- Summary blurb (enter in submission system): A short text summarizing in a single sentence the study (max. 200 characters including spaces). This text is used in conjunction with the titles of papers, hence should be informative and complementary to the title. It should describe the context and significance of the findings for a general readership; it should be written in the present tense and refer to the work in the third person. Author names should not be mentioned.

The license to publish form must be signed before your manuscript can be sent to production. A link to the license to publish form will be available to the corresponding author only. Please take a moment to check your funder requirements.

Reviews, decision letters, and point-by-point responses associated with peer-review at Life Science Alliance will be published, alongside the manuscript. If you do want to opt out of having the reviewer reports and your point-by-point responses displayed, please let us know immediately.

Thank you for your attention to these final processing requirements. Please revise and format the manuscript and upload materials as soon as you are able.

Thank you for this interesting contribution to the literature. We look forward to publishing your paper in Life Science Alliance.

Sincerely,

Tim Fessenden
Scientific Editor
Life Science Alliance

October 28, 2026

RE: Life Science Alliance Manuscript #LSA-2026-03939-TR

Dr. Tomoki Kuwahara
The University of Tokyo
Dept. Neuropathology, Grad. Sch. Med.
7-3-1, Hongo, Bunkyo-ku
Tokyo 1130033
Japan

Dear Dr. Kuwahara,

Thank you for submitting your Research Article entitled "PIKfyve inhibition induces LRRK2 activation through phosphatidylinositol 3-phosphate and CASM". It is a pleasure to let you know that your manuscript is now accepted for publication in Life Science Alliance. Congratulations on this interesting work.

The final published version of your manuscript will be deposited by us to PubMed Central upon publication.

Your manuscript will now progress through copyediting and proofing. It is journal policy that authors provide original data upon request.

Your article will publish open access upon publication under a CC-BY license.

Reviews, decision letters, and point-by-point responses associated with peer-review at Life Science Alliance will be published, alongside the manuscript. If you do want to opt out of having the reviewer reports and your point-by-point responses displayed, please let us know immediately.

IMPORTANT: If you will be unreachable at any time, please provide us with the email address of an alternate author. Failure to respond to routine queries may lead to unavoidable delays in publication.

Scheduling details will be available from our production department. You will receive proofs shortly before the publication date. Only essential corrections can be made at the proof stage so if there are any minor final changes you wish to make to the manuscript, please let the journal office know now.

DISTRIBUTION OF MATERIALS:
Authors are required to distribute freely any materials used in experiments published in Life Science Alliance. Authors are encouraged to deposit materials used in their studies to the appropriate repositories for distribution to researchers.

You can contact the journal office with any questions, contact@life-science-alliance.org

Again, congratulations on a very nice paper. I hope you found the review process to be constructive and are pleased with how the manuscript was handled editorially. We look forward to future exciting submissions from your lab.

Sincerely,

Tim Fessenden
Scientific Editor
Life Science Alliance
